# The value for money of artificial intelligence-empowered precision medicine: a systematic review and regression analysis

**DOI:** 10.1038/s41746-025-02259-w

**Published:** 2025-12-24

**Authors:** Yue Zhang, Ziwei Lin, Yot Teerawattananon, Katika Akksilp, Alec Morton, Yi Wang, Thittaya Prapinvanich, Thamonwan Dulsamphan, Wenjia Chen

**Affiliations:** 1 Saw Swee Hock School of Public Health, National University of Singapore, Singapore, Singapore; 2 Department of Emergency Medicine, Sengkang General Hospital, Singapore, Singapore; 3 Health Intervention and Technology Assessment Program (HITAP), Ministry of Public Health, Nonthaburi, Thailand; 4 Institute of Medical Research and Technology Assessment (IMRTA), Ministry of Public Health, Nonthaburi, Thailand; 5 Department of Management Science, Strathclyde Business School, University of Strathclyde, Glasgow, UK; 6 Yale-NUS College, Singapore, Singapore

**Keywords:** Health care economics, Health services

## Abstract

Artificial intelligence has empowered precision medicine (AI-PM) to transform healthcare. This study synthesized available evidence on the cost-effectiveness of AI-PM. We systematically searched five major databases for economic evaluations of AI-PM, extracted data, and assessed risk-of-bias using the Bias in Economic Evaluation (ECOBIAS) checklist. For cost-utility analyses, the value-for-money was quantitatively summarized, and regression analyses incorporating machine learning were conducted to explore value heterogeneity. Forty-eight economic evaluations were included, of which 31 were cost-utility analyses. Although risk-of-bias assessment indicated potential systematic optimism, AI-PM was cost-saving or cost-effective in 89% of base-case analyses, with incremental cost-effectiveness ratios ranging from dominant to $129,174/quality-adjusted life-year (QALY). Interquartile ranges of incremental costs (−$259 to $28), QALY gains (0.001–0.019), and net monetary benefits (NMB; $18 to $986 at a willingness-to-pay threshold equal to one-time per-capita GDP) indicated modest health gains at minimal additional costs, and likely high value heterogeneity. Modeling choices and system-level factors were identified as essential sources of heterogeneity in estimated NMBs. Additional value assessment revealed low adaptability and underreported key value factors, leaving significant uncertainties in AI-PM adoption.

## Introduction

Precision medicine (PM) offers a promising approach that tailors medical decisions, treatments, and interventions to an individual’s unique characteristics, encompassing genomic, imaging, clinical, social/behavioral, and environmental data^1,2^. In this digital era, advancements in artificial intelligence (AI) and big health data can further empower PM, integrating diverse data sources at an enormous scale to recognize sophisticated patterns and hidden relationships with exceptional accuracy^3,4^. AI is an umbrella term comprising a diverse set of technologies that leverage advanced computation and inference to mimic human intelligence. For instance, machine learning enables systems to identify patterns, make predictions, and adapt by learning from data, using techniques such as penalized regressions, neural networks, and deep learning^5,6^. Machine learning can be combined with rule-based algorithms, natural language processing, generative AI, and IT technologies to support the development of a wide range of data-driven healthcare interventions to advance PM. These applications of AI in the PM domain can be referred to as AI-empowered PM or “AI-PM”.

According to Davenport and colleagues^7^, AI-PM tools can be classified into four major types: (1) *digital diagnostics*, which typically combines deep learning with 3D technologies to enhance the imaging diagnostics of various diseases, such as the Heartflow FFRCT^8^, SELENA + ^9^, and CureMetrix^10^; (2) *risk prediction*, which applies AI algorithms to predict disease disposition and progression for healthcare triage or treatment escalation, such as Singapore’s AI-assisted 3H Care System^11^; (3) *precision treatment*, which prioritizes therapeutic options from each patient’s genetic profiles on drug susceptibility or responses, such as CureMatch^12^, CancerIQ^13^, and Oncobox^14^; and (4) *disease self-management*, which connects AI algorithms to self-monitoring medical and treatment devices to empower disease self-control, such as the DBLG1 system^15^. Beyond our study scope of AI-PM, AI-based digital health can also be used for patient management (e.g., education, medication reminders, patient journey), drug discovery, and administrative processes, which do not involve patient-tailored decisions based on their particular risk profiles.

Although AI-PM is rapidly evolving and demonstrates bright potential in improving healthcare delivery and outcomes, due to lagging clinical evidence and scarce economic analyses, its implementation in real-world settings remains limited^16,17^. The potential of AI-PM cannot be realized without a comprehensive assessment of its clinical effectiveness and economic implications. Economic evaluation (EE) provides a systematic framework for assessing the estimated costs relative to expected outcomes, thereby informing the value for money of health technologies^18^. In particular, EE conducted at the market-access stage aims to help regulators, clinicians, and payers in making decisions on clinical adoption, pricing strategies, and reimbursement^19^. Early EE provides guidance to researchers, manufacturers, and investors on research priorities, product development, and management of uncertainties during the developmental phase^20^.

AI-PM, the subcategory of AI-based health interventions, has reached maturity only recently and requires further proof of its economic value for wider application. Two recent systematic reviews, conducted by Voets et al. and Vithlani et al., have narratively summarized 20 and 21 EEs on AI-based health interventions, focusing on study characteristics and cost-effectiveness outcomes^21,22^. However, these reviews neither quantified evidence into common metrics for comparison nor identified sources of value heterogeneity. Additionally, potential study biases remained unassessed. Therefore, our study builds on and extends previous work by addressing existing knowledge gaps. Specifically, it quantifies the cost-effectiveness profiles of different types of AI-PM tools across various contexts and conditions, identifies sources of value heterogeneity, and assesses potential biases.

## Results

### Literature search

**Fig. 1:**
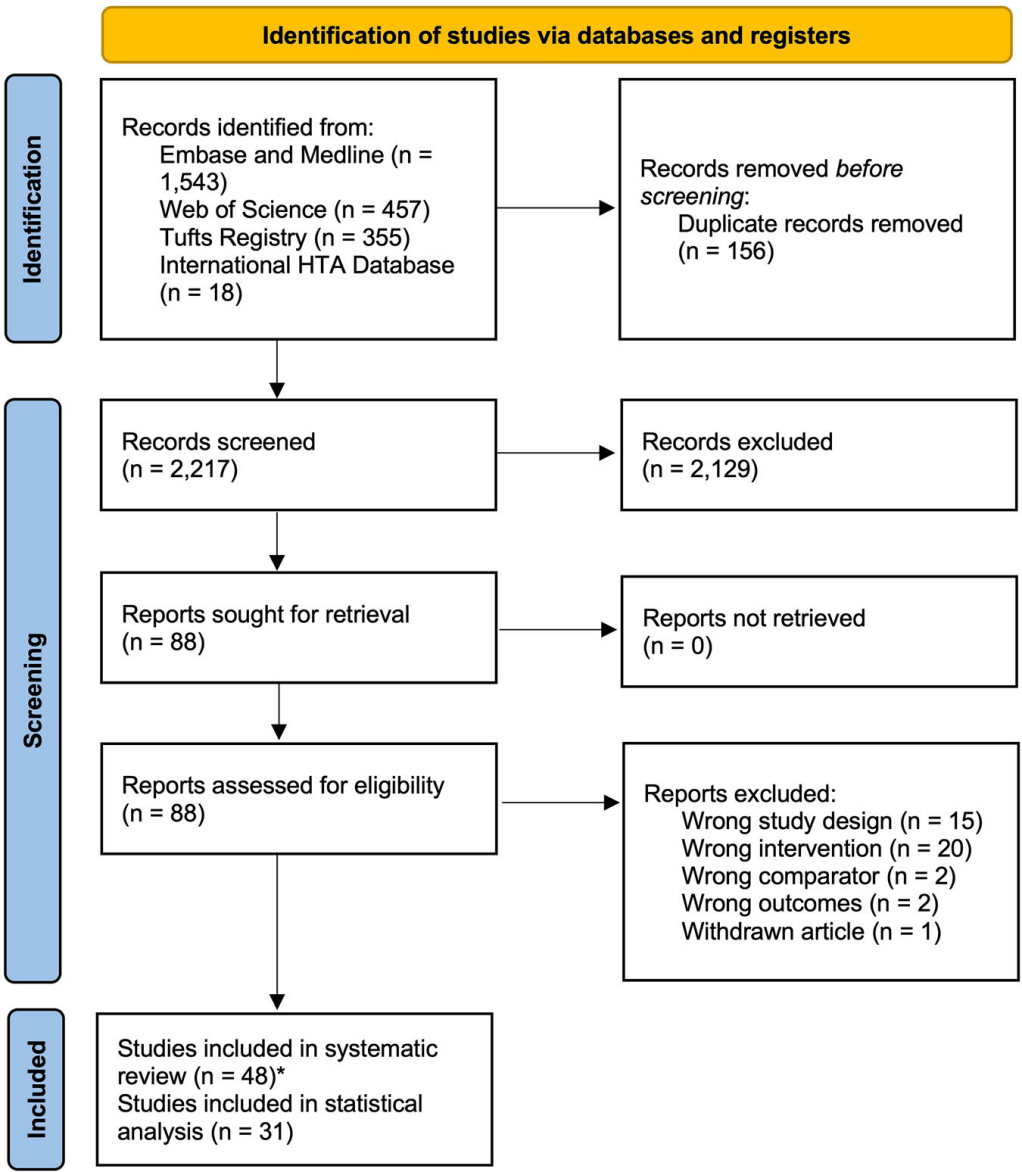
PRISMA flowchart of included studies. *The number of study records is 48 for the final inclusion, resulting in a total of 50 datasets; this is because one study analyzed 3 AI-PM interventions, which was accounted for thrice.

Figure 1 shows the Preferred Reporting Items for Systematic Reviews and Meta-Analyses (PRISMA) flowchart of study selection. A total of 2373 papers were identified from the initial systematic search, of which 48 papers were selected for data extraction and quality assessment (Supplementary Table 1). Of note, the number of EEs on AI-PM has increased sharply between 2022 and 2023 (Supplementary Fig. 1).

### Characteristics of EEs of AI-PM

The study characteristics, methodological aspects, and AI-PM features of the 48 studies (totaling 50 datasets) are presented in Table 1, with detailed information by AI-PM types available in Supplementary Table 2.

**Table 1:** Characteristics of included studies (*N* = 50^a^)

Category	Subcategory	*N* (%)
**Study characteristics**		
Country/region of analysis	United States of America	19 (38.0)
	United Kingdom	3 (6.0)
	Canada	3 (6.0)
	China	5 (10.0)
	Germany	4 (8.0)
	Pakistan	2 (4.0)
	Singapore	2 (4.0)
	Sweden	2 (4.0)
	Others^b^	10 (20.0)
Target population—age	Pediatric	4 (8.0)
	Adult	26 (52.0)
	Senior	5 (10.0)
	All ages	6 (12.0)
	Not specified	9 (18.0)
Target population—sex	All male	0 (0)
	All female	3 (6.0)
	Gender/sex-neutral	47 (94.0)
Disease domain (ICD-11 category)	Certain infectious or parasitic diseases	7 (14.0)
	Diseases of the circulatory system	3 (6.0)
	Diseases of the digestive system	5 (10.0)
	Diseases of the nervous system	2 (4.0)
	Diseases of the visual system	12 (24.0)
	Injury, poisoning, or external causes	3 (6.0)
	Mental, behavioral or neurodevelopmental disorders	3 (6.0)
	Neoplasms	12 (24.0)
	Others^c^	3 (6.0)
Funder type	Public	16 (32.0)
	Private—not for profit	3 (6.0)
	Private—for profit	9 (18.0)
	Mixed funding source	6 (12.0)
	No funding source	8 (16.0)
	Not stated	8 (16.0)
Developmental stage	Early stage	10 (20.0)
	Market-access stage	40 (80.0)
**Model specifications**		
Economic evaluation type	Budget impact analysis	6 (12.0)
	Cost minimization analysis	3 (6.0)
	Cost effectiveness analysis	10 (20.0)
	Cost utility analysis	28 (56.0)
	Cost effectiveness analysis and cost utility analysis	3 (6.0)
Model type	Modeling—Markov	15 (30.0)
	Modeling—decision tree	11 (22.0)
	Hybrid model	12 (24.0)
	Patient-level simulation	4 (8.0)
	Other type of analysis (non-model)	8 (16.0)
Time horizon	Short term (≤3 years)	14 (28.0)
	Intermediate term (3–10 years)	10 (20.0)
	Long term (10–30 years)	2 (4.0)
	Lifetime (>30 years)	23 (46.0)
	Not applicable	1 (2.0)
Study perspective	Societal	9 (18.0)
	Health system	38 (76.0)
	Patient	1 (2.0)
	Not specified	2 (4.0)

Intervention characteristics		
Type of AI-PM tool	Digital diagnostics	33 (66.0)
	Risk prediction	13 (26.0)
	Precision treatment	1 (2.0)
	Disease self-management	3 (6.0)
Type of digital/electronic product	Software	34 (68.0)
	Interactive application	3 (6.0)
	Device	1 (2.0)
	Standalone algorithm	12 (24.0)
Integration of AI performance rate	Yes	40 (80.0)
	No	10 (20.0)
Integration of adherence to AI technology	Yes	3 (6.0)
	No	47 (94.0)
Integration of compliance with AI-informed decisions	Yes	6 (12.0)
	No	44 (88.0)
Direct reporting of AI-PM costs	Yes	43 (86.0)
	No	7 (14.0)
AI-PM unit cost, (median [interquartile range])	$10.20 ($1.12 to $51.76)	
Provided detailed specifications of AI-PM costs	Yes	16 (32.0)
	No	34 (68.0)

*AI* artificial intelligence, *ICD* international classification of diseases, *PM* precision medicine.

^a^*N* = 50 as one study analyzed 3 AI-PM interventions, which was accounted for thrice, resulting in a total of 50 datasets in base-case descriptive analysis.

^b^The subcategory “Others” included 1 study from Australia, 1 study from Brazil, 1 study from Belgium, 1 study from Italy, 1 study from Japan, 1 study from Malawi, 1 study from Spain, 1 study from Taiwan, 1 study from Thailand, and 1 study from the Netherlands.

^c^The subcategory “Others” included 1 study under diseases of the genitourinary system, 1 study under diseases of the musculoskeletal system or connective tissue, and 1 study under not specified.

Among the 50 datasets, there were 28 cost-utility analyses (CUAs, 56%), 10 cost-effectiveness analyses (CEAs, 20%), 6 budget impact analyses (BIAs, 12%), and 3 cost-minimization analyses (CMAs, 6%). Three EEs (6%) presented findings in both CEAs and CUAs. Notably, the majority were EEs at the market-access stage (80%), while the remaining 20% were early EEs, with risk prediction tools showing the highest proportion of early evaluations among AI-PM types (38.4%).

Most EEs were conducted in the United States (38%), China (10%) and Germany (8%), with 32% sponsored by public agencies, 18% by private-for-profit agencies, 6% by private not-for-profit agencies, while the rest received mixed funding, no funding, or did not specify funding sources. Additionally, over half of the evaluated interventions targeted adults (52%) and were gender/sex-neutral (94%), with the majority applying to cancer (24%) and visual diseases (24%).

The most common AI-PM type was digital diagnostics (66%), followed by risk prediction tools (26%). Only three studies (6%) evaluated disease self-management tools, and one study (2%) evaluated precision treatment tools. Software was the most evaluated form of digital/electronic products (68%), followed by algorithms alone (24%), interactive web/phone applications (6%), and interconnected devices (2%). Stratified evidence showed that 84.8% of digital diagnostics were software-based, whereas for the risk prediction tools, nearly half of the products relied on standalone algorithms (46.2%). AI performance, adherence to AI technology, and compliance with AI-informed decisions were integrated in 40 (80%), 3 (6%), and 6 (12%) of 50 datasets, respectively. AI-PM costs were directly reported in 43 datasets, with a median cost per person of $10.20 (range: $0 to $3475.96, interquartile range [IQR]: $1.12 to $51.76), although only 32% (*N* = 16) provided a detailed specification of cost components.

A lifetime horizon (46%) and health system perspective (76%) were most frequently applied. In particular, 84% of the datasets applied modeling approaches, while the remaining 16% were based on pragmatic trials or impact studies.

### Value for money of AI-PM

The value for money of AI-PM was summarized from 108 cost-effectiveness estimates of 31 CUAs, including both base-case and sensitivity analyses.

**Fig. 2:**
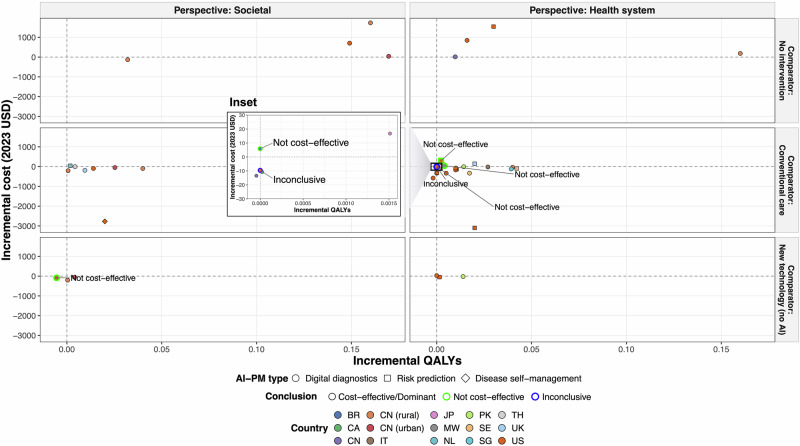
Common cost-effectiveness plane for incremental costs (2023 United States dollars [USD]) and incremental QALYs from different perspectives and comparators. AI artificial intelligence, QALYs quality-adjusted life-years, PM precision medicine, BR Brazil, CA Canada, CN China, IT Italy, JP Japan, MW Malawi, NL Netherlands, PK Pakistan, SE Sweden, SG Singapore, TH Thailand, UK United Kingdom, US United States. Results from the base-case analyses of included cost-utility analyses were extracted for AI-PM interventions compared against different types of comparators and from different perspectives. When multiple cost-effectiveness estimates were reported for the same comparator type and perspective within a study, the median value was used. Sensitivity analysis results were excluded. Perspectives were categorized as societal or health system, and comparators as no intervention (e.g., natural history, no screening), conventional care (e.g., manual screening), and new technology without AI (e.g., telemedicine, genomic PM). The inset shows a magnified view of the area marked by the black rectangle, i.e., ICER points within the range of incremental QALYs from −0.00015 to 0.0016 and incremental costs from −$30 to $30.

Figure 2 presents the base-case incremental costs (Δcosts) plotted against incremental quality-adjusted life-years (ΔQALYs) across different perspectives and comparators in a common cost-effectiveness plane. Overall, 26 (56%) and 15 (33%) out of 46 incremental cost-effectiveness ratios (ICERs) for any AI-PM intervention type were found to be cost-saving and cost-effective, respectively, under the original willingness-to-pay (WTP) thresholds, ranging from dominant to $129,174/QALY. One study (2%) did not draw a definitive conclusion due to no QALY difference. The four unfavorable cost-effective results (9%) included three evaluations of digital diagnostics in low- and middle-income countries (Brazil, China, and Malawi) and one risk prediction tool evaluated in the United States. When stratified by perspective and comparator type, most non-cost-effectiveness (three out of four) were from studies adopting a health system perspective with conventional care (e.g., manual screening) as the comparator, while the remaining one was reported from a societal perspective comparing against a new technology without AI (e.g., telemedicine or genomic precision medicine).

**Fig. 3:**
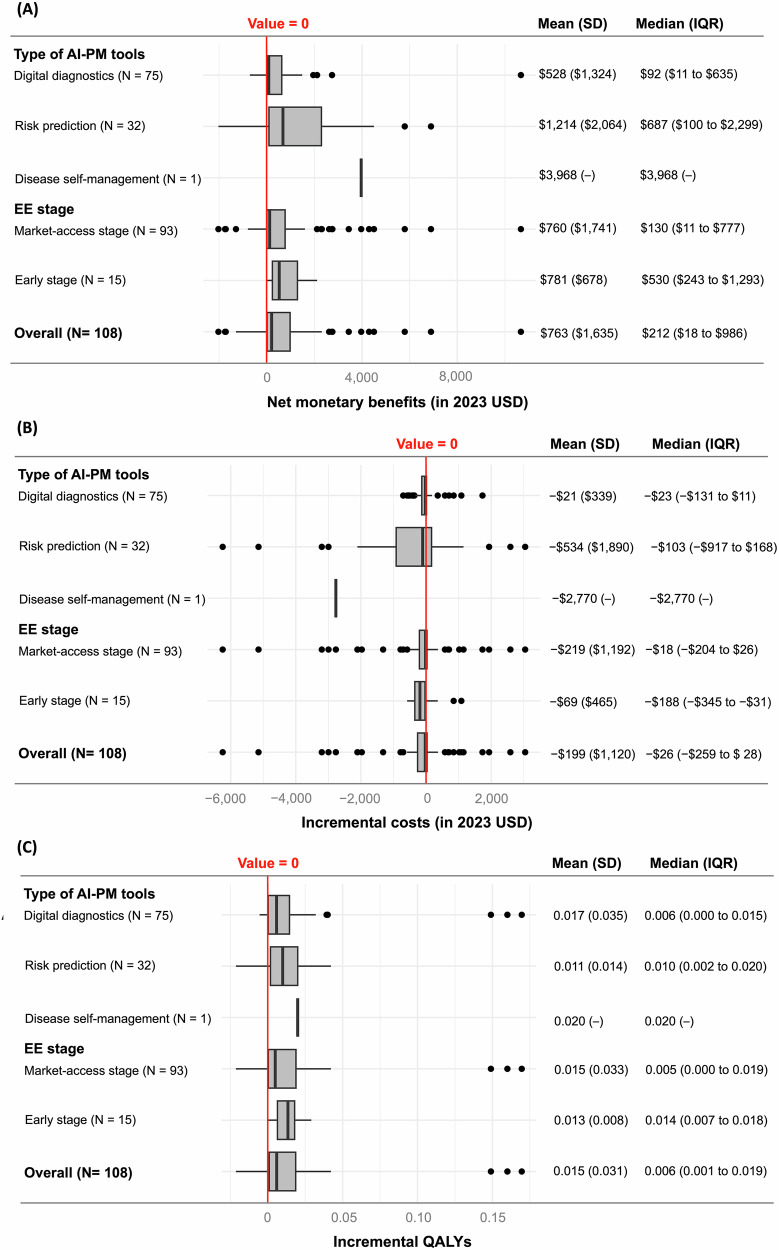
Summary boxplots showing the distribution of cost-effectiveness of AI-PM interventions. **A** Net monetary benefits. **B** Incremental costs. **C** Incremental QALYs. AI artificial intelligence, EE economic evaluation, QALYs quality-adjusted life-years, IQR interquartile range, SD standard deviation, PM precision medicine. The box indicates the interquartile range (IQR), with the center line denoting the median. IQR refers to the range between the first quartile (25th percentile) and the third quartile (75th percentile), representing the middle 50% of the data. Whiskers extend to the most extreme values within 1.5 × IQR beyond the quartiles, and outliers are shown as individual dots. The vertical red line refers to the value of 0.

The distributions of Δcosts, ΔQALYs, and net monetary benefits (NMBs) are further displayed in Fig. 3. Overall, Δcosts ranged between −$6245 and $3044, with a median of −$26 (IQR = −$259 to $28) and a mean of −$199 (standard deviation [SD] = $1120). ΔQALYs ranged between −0.021 and 0.17, with a median of 0.006 (IQR = 0.001–0.019) and a mean of 0.015 (SD = 0.031). NMBs ranged between −$2022 and $10,669, with a median of $212 (IQR = $18 to $986) and a mean of $763 (SD = $1,635). Of note, early-stage EEs tended to report a higher median NMB compared to EEs at the market-access stage, but there was no statistical difference (median [IQR]: $530 [$243 to $1293] vs. $130 [$11 to $777], *p* = 0.127). In addition, stratified by AI-PM types, risk prediction tools had a higher median NMB compared to digital diagnostics (median [IQR]: $687 [$100 to $2299] vs. $92 [$11 to $635], *p* = 0.010), potentially driven by the former’s lower Δcost (median [IQR]: −$103 [−$917 to $168] vs. −$23 [−$131 to $11], *p* = 0.320) and higher ΔQALY (median [IQR]: 0.01 [0.002–0.020] vs. 0.006 [0.000 to 0.015], *p* = 0.307). Meanwhile, only one CUA of AI-PM focused on disease self-management, reporting an NMB of $3968.

The top five AI-PM tools in terms of NMBs (Supplementary Table 3) were digital diagnostics for polyp identification in colonoscopy (NMB = $10,669)^23^, risk prediction for pre-surgical opioid use disorder (NMB = $4504)^24^, automated tuberculosis treatment monitoring (NMB = $3968)^25^, risk prediction for stratified breast cancer screening (NMB = $3447)^26^, and digital diagnostics for diabetic retinopathy detection (NMB = $2745)^27^. The top five AI-PM tools by the lowest Δcosts and the highest ΔQALYs are additionally provided in Supplementary Table 3, with risk prediction tools and digital diagnostics being the most frequent, respectively. All these tools were also found to be dominant based on ICERs in the base-case analyses.

### Potential sources of value heterogeneity

Supplementary Table 4 provides the results of univariate mixed-effects regression analyses, where 6, 4, and 7 variables had *p* values < 0.1 among the 17 candidate predictors for NMBs, Δcosts, and ΔQALYs, respectively. A penalized Lasso regression was then performed to identify essential value drivers for the three outcomes. Table 2 presents the multivariate mixed-effects regression results based on Lasso-selected essential predictors for NMBs, Δcosts, and ΔQALYs.

**Table 2:** Lasso regression results on NMBs, Δcosts, and ΔQALYs (*N* = 108^a^)

Variables	Coefficient	95% CI	*P* value
**Dependent variable: NMBs**			
Country income level			
Low or middle income	(Reference)		
High income	776	[−142, 1693]	0.097
Funder type			
Public or private-not-for-profit	(Reference)		
Private-for-profit	248	[−972, 1469]	0.690
No/unspecified funding sources	−520	[−1343, 302]	0.215
AI-PM unit cost	**2.94**	**[1.65, 4.23]**	**0.000**
Choice of comparators			
Current practice/standard of care/none	(Reference)		
New technology/best alternative/major competitor	**−665**	**[−1157**, **−174]**	**0.008**
Integration of compliance with AI-informed decisions			
No	(Reference)		
Yes	−1200	[−2821, 421]	0.147
Study perspective			
Societal	(Reference)		
Health system	−1299	[−2642, 43]	0.058
Lifetime horizon			
No	(Reference)		
Yes	−317	[−916, 282]	0.300
**Random part of the model:**	**Component**	**Variance**	**Standard Deviation**
	Random intercept	7.23 × 10^−30^	2.69 × 10^−15^
	Residual	1,803,776	1343
**Dependent variable: Δcosts**			
Funder type			
Public or private-not-for-profit	(Reference)		
Private-for-profit	−294	[−936, 347]	0.368
No/unspecified funding sources	140	[−248, 528]	0.480
AI-PM unit cost	**−2.46**	**[−3.37**,**−1.54]**	**0.000**
Integration of AI performance			
No	(Reference)		
Yes	813	[−323, 1948]	0.161
Integration of compliance with AI-informed decisions			
No	(Reference)		
Yes	1274	[−573, 3121]	0.176
Lifetime horizon			
No	(Reference)		
Yes	349	[−60, 757]	0.094
**Random part of the model:**	**Component**	**Variance**	**Standard Deviation**
	Random intercept	2.36 × 10^−30^	1.54 × 10^−15^
	Residual	867,154	931
**Dependent variable: ΔQALYs**			
Target population—age			
All/not specified	(Reference)		
Pediatric	−0.002	[−0.015, 0.010]	0.715
Adult	−0.008	[−0.019, 0.002]	0.127
Senior	−0.009	[−0.023, 0.004]	0.168
Target population—sex			
Gender/sex-neutral	(Reference)		
All male/all female	−0.004	[−0.010, 0.001]	0.109
Choice of comparators			
Current practice/standard of care/none	(Reference)		
New technology/best alternative/major competitor	**−0.017**	**[−0.034**, **−0.001]**	**0.042**
Integration of adherence to AI technology			
No	(Reference)		
Yes	**0.045**	**[0.005, 0.085]**	**0.027**
Applied any EE guidance/reference case			
No	(Reference)		
Yes	**−0.010**	**[−0.016**,**−0.004]**	**0.002**
Study perspective			
Societal	(Reference)		
Health system	−0.010	[−0.043, 0.023]	0.543
**Random part of the model:**	**Component**	**Variance**	**Standard Deviation**
	Random intercept	6.26 × 10^−38^	2.50 × 10^−19^
	Residual	6.24 × 10^−4^	0.025

All currency is presented in 2023 United States dollars. Bold values indicate statistically significant results (*p* < 0.05).

*AI* artificial intelligence, *CI* confidence interval, *Δcosts* incremental costs, *ΔQALYs* incremental quality-adjusted life-years, *EE* economic evaluation, *NMBs* net monetary benefits, *PM* precision medicine.

^a^*N* = 108 as the analysis sample for the mixed-effects regression models comprised 108 cost-effectiveness estimates of 31 cost-utility analyses.

For NMBs, essential fixed-effect predictors were country income level, funder type, unit cost of AI-PM, integration of compliance with AI-informed decisions, choice of comparators, study perspective, and time horizon. Particularly, $1 increase in AI-PM unit cost would lead to a $2.94 increase in NMB (95% confidence interval [CI]: 1.65–4.23). Additionally, when the comparator was the best alternative (e.g., telemedicine, genomic PM) rather than the standard of care (SoC), NMB would decrease by $665 (95% CI: −1157 to −174). Meanwhile, the random intercept standard deviation was estimated at 2.69 × 10^−15^, whereas the residual within-study standard deviation was 1343, indicating that substantial uncaptured variability remained after covariate adjustment, most of which resided within studies rather than between studies.

For Δcosts, essential fixed-effect factors included funder type, AI-PM unit cost, integration of AI performance, integration of compliance with AI-informed decisions, and lifetime horizon. In particular, a $1 increase in AI-PM unit cost was associated with a $2.46 reduction in Δcost (95% CI: −3.37 to −1.54). The random intercept standard deviation was 1.54 × 10^−15^, whereas the residual within-study standard deviation was 931.

For ΔQALYs, target population age, target population sex, choice of comparators, integration of adherence to AI technology, usage of EE guidance/reference case, and study perspective were identified as essential fixed-effect predictors. In particular, ΔQALY significantly decreased when the comparator was the best alternative rather than SoC, and when following EE guidance/reference case. Additionally, the random intercept standard deviation was 2.50 × 10^−19^, whereas the residual within-study standard deviation was 0.025.

### Risk-of-bias assessment in model-based EEs

**Fig. 4:**
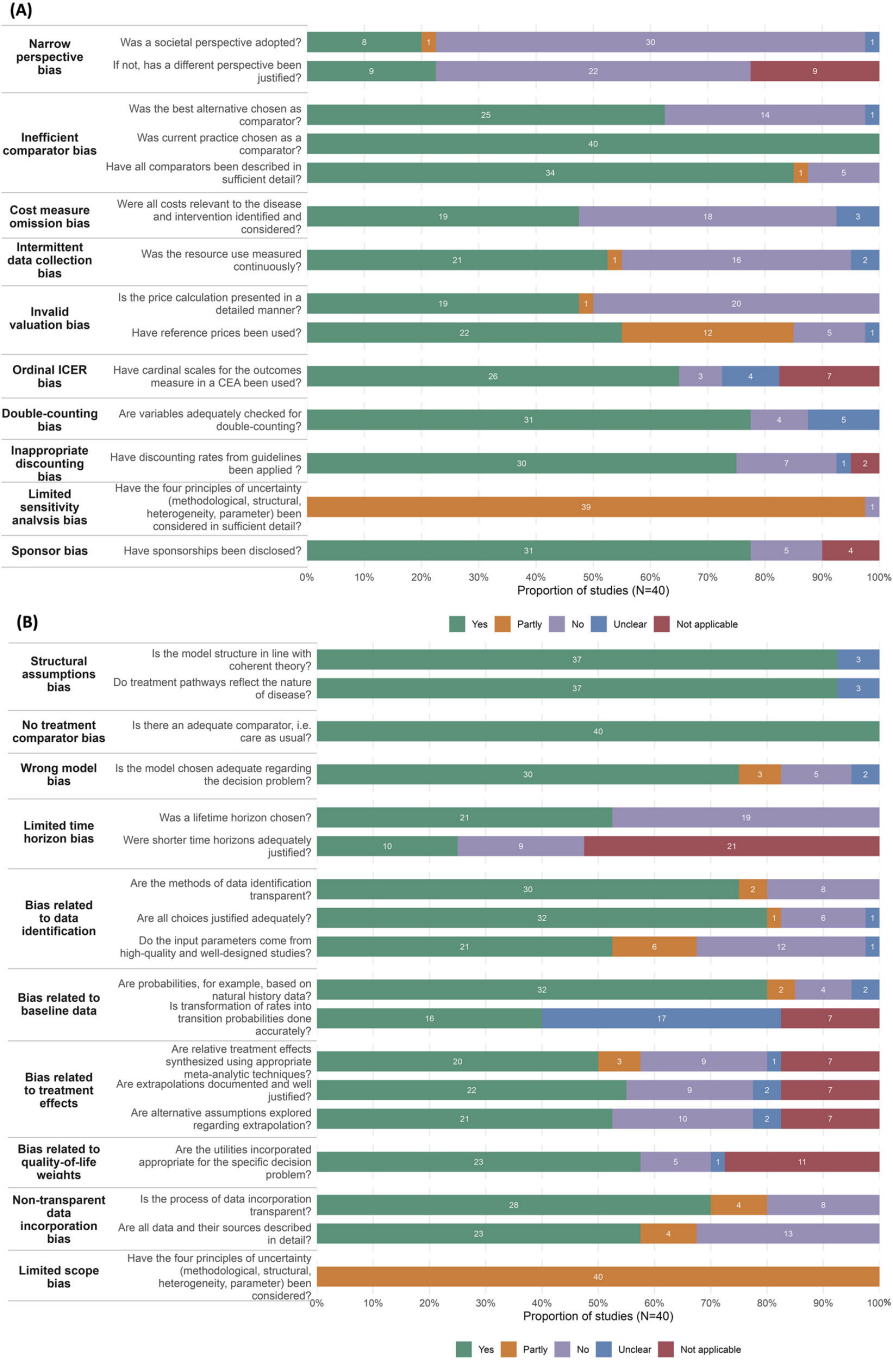
Proportion of studies reporting ECOBIAS assessment items. **A** Overall checklist for bias in economic evaluation. **B** Model-specific aspects of bias in economic evaluation. *N* = 40 as 8 non-model studies were not eligible. Yes—low risk of bias; Partly—partly risk of bias; No—high risk of bias.

In the Bias in Economic Evaluation (ECOBIAS) assessment of 40 model-based studies (Fig. 4), 32 detailed items were critically appraised. Overall, seven items were graded as high risk of bias in over one-third of the studies. These included narrow perspective: non-societal perspective (75.0%) and lack of justification (55.0%); invalid valuation: insufficient presentation of price calculations (50.0%); limited time horizon: non-lifetime horizon (47.5%); cost measure omission (45.0%); intermittent data collection (40.0%); and inefficient comparator: best alternative not chosen (35%).

### Additional value and remaining uncertainty surrounding clinical adoption

Table 3 summarizes the additional value assessment of AI-PMs across all 48 included EEs. The majority of evaluations clearly reported key aspects of the use case validity, including clinical pathway (98%), intervention operation (75%), and decision rationale (65%). Implementation requirements mainly covered medical devices or specific setups (63%) but overlooked smart devices (15%) or internet connectivity (10%), and training needs for physicians (17%) and patients (6%) were often not addressed. Regarding ethical and legal considerations, while 15 (31%) EEs discussed ethical issues of AI-PM development and deployment, little attention was given to data security compliance (2%) and liability associated with AI-informed decisions (0%). Other value factors critical for the responsible adoption of AI-PM, such as usability (<5%), user safety concerns (0%), and patient empowerment (6%), were also rarely evaluated. Additionally, most evaluated AI-PM tools were either not adaptable (31%) or their adaptability to different settings remained unclear (63%).

**Table 3:** Additional value assessment of AI-PM (*N* = 48)

Domain	Additional value/barriers		*N* (%)
Patient/user experience	Discussed how AI-PM engaged and/or empowered patients		3 (6.3)
Use case validity	Clarified AI-involved clinical pathway		47 (97.9)
	Justified the AI-involved clinical pathway		47 (97.9)
	Described the operation of AI intervention		36 (75.0)
	Discussed the rationale of AI-driven decisions		31 (64.6)
Technical readiness	Discussed the implementation requirements	Smart device	7 (14.6)
		Internet connection	5 (10.4)
		Medical devices or setups	30 (62.5)
		Patient training	3 (6.3)
		Physician/operator training	8 (16.7)
	Usability assessment		2 (4.2)
	Any user safety concerns and monitoring		0 (0)
Ethical and legal considerations	Data security compliance		1 (2.1)
	Reported liability aspects of AI-informed clinical decision-making		0 (0)
	Reported ethical issues		15 (31.3)
Adaptability	Current evidence is generalizable to a broader or different population	Yes	3 (6.3)
		No	15 (31.3)
		Unclear	30 (62.5)

*AI* artificial intelligence, *PM* precision medicine.

## Discussion

While previous reviews only provided descriptive summaries of EEs of AI-based health interventions^21,22^, our study was the first to quantify the value for money of AI-PM and identify essential drivers of value heterogeneity. In the 48 included EEs, evaluated AI-PM tools were mainly targeted at adults without sex specification, applied to eye diseases or neoplasms, and used for digital diagnostics or risk prediction. ICERs from the base-case analyses indicated that most (89%) AI-PM interventions were cost-saving or cost-effective, ranging from dominant to $129,174/QALY, with non-cost-effective results mainly for digital diagnostics evaluated in resource-constrained settings from a health system perspective. Across studies, AI-PM generally achieved modest QALY gains (median [IQR]: 0.006 [0.001–0.019]) at minimal additional costs or even cost-saving (median [IQR]: −$26 [−$259 to $28]). Correspondingly, NMBs overall (median [IQR]: $212 [$18 to $986]) tilted toward being cost-effective but had high heterogeneity. Notably, although the median NMBs appeared higher in risk prediction tools compared to digital diagnostics ($687 vs $92), accompanied by much higher proportions of early EEs and standalone AI algorithms with lower unit costs in the former, this comparison should be interpreted with caution because the former also showed wider variability (IQR: $100 to $2299 vs. $11 to $635) while medians can mask extreme variability. Meanwhile, evidence on precision treatment tools and disease self-management tools remained scarce and inconclusive. Nonetheless, AI-PM’s value-for-money estimates were strongly associated with modeling choices, country income levels, and funder types, suggesting potential systematic bias, which has been confirmed by the ECOBIAS assessment. Moreover, most evaluated AI-PM tools had low or unclear adaptability and often underreported key value factors such as technical readiness, ethical and legal considerations, and other patient/user aspects, which may pose additional barriers to clinical adoption.

Compared to genomic PM in a previous systematic review^28^, data-driven AI-PM tools overall showed a higher proportion of studies being dominant or cost-effective (89% vs. 67%) and tended to have higher NMBs under the same unified WTP threshold equal to one-time gross domestic product (GDP) per capita (median [IQR]: $212 [$18 to $986] vs. $135 [unreported]), but lower QALY gains (median [IQR]: 0.006 [0.001–0.019] vs. 0.05 [unreported]), likely due to AI-PM’s role as decision support tools rather than primary or companion diagnostics. Unlike sequencing technologies that identify specific biological markers to precisely diagnose disease predispositions or determine treatment responses, AI-driven predictors rely on existing real-world data to estimate risks and guide decision-making. Thus, the effectiveness of AI-PM is influenced by technical performance and the clinical impact of AI-informed decisions, which are in turn influenced by data quality and availability, model generalizability, physicians’ risk preferences, practice habits, and attitudes toward AI-PM alerts, as well as other external factors^29^, potentially introducing high heterogeneity into measurable health gains and leading to a lower median. While the health benefits of AI-PM were considered modest, QALYs alone may not capture the broader value of these interventions, such as patient empowerment (e.g., well-being, independence, self-control) and workflow efficiency (e.g., prompt alert and feedback, shared decision-making)^21,30^. Moreover, through self-learning and retraining, AI models can update themselves to improve performance, whose value was often overlooked. A comprehensive assessment incorporating these additional value elements could provide a more complete understanding of AI-PM’s benefits.

Since the release of ChatGPT in November 2022^31^, advancements in generative AI have rapidly accelerated the evolution of AI-PM. Recent developments include the integration of generative models into digital twins for patient simulation^32^, health trajectory forecasting^33^, and patient-facing conversational systems^34^. In parallel, digital health technologies (DHTs) are becoming increasingly modular and interconnected^35^, forming flexible suites of networked devices and cloud services that collaboratively deliver care across settings. Together, these generative AI and modular DHT innovations are reshaping how personalized care is organized, evaluated, and reimbursed, driving the development of a new generation of AI-PM interventions. One notable emerging example is the “personal health agent”^36^, which leverages generative AI within multi-agent systems to provide continuous, context-aware, and personalized health guidance. Such transformative changes in AI-PMs towards interactive, generative, and multimodal paradigms introduce new complexities in the concept and methodology of EEs of AI-PM, which are not covered in the current review because they need separate investigation.

Given the substantial value heterogeneity, we adopted a multi-dimensional mixed-effects approach to identify and adjust for their sources, with regression analyses conducted separately for three economic dimensions (Δcosts, ΔQALYs, and NMBs). For each outcome, the regressions incorporated a comprehensive set of 17 covariates representing disease, contextual, methodological, and intervention factors to address observed heterogeneity, while the mixed-effects model specification allowed unobserved between-study heterogeneity to be modeled as random effects. After LASSO regularization, two intervention parameters (AI-PM unit costs, compliance with AI-informed decisions) were retained as essential predictors of AI-PM’s NMBs. Interventions with higher AI-PM unit costs were found to have lower Δcosts and greater NMBs. Across AI-PM types (see scatter plots in Supplementary Fig. 2), while the association between unit costs and NMBs was not significant (or possibly slightly negative) for digital diagnostics, a high-cost “omics-based” risk prediction tool ($597/unit) for classifying opioid use disorder risk in analgesic prescribing showed exceptionally high NMBs (base-case: $4298 to $4504)^24^. This may reflect the impact of advanced genomic classification in improving accuracy, efficiency, and cost-effectiveness. On the other hand, NMBs decreased when compliance with AI-informed decisions was integrated into the model, indicating the significance of this parameter in yielding more realistic and less biased estimates of AI-PM’s true value.

Beyond the intervention pathway, modeling choices (perspective, time horizon, comparator) and system-level factors (country income level, study sponsor) were also retained as essential predictors, explaining bias in NMBs. In line with general EE evidence, SoC as the comparator yielded $665 higher NMBs than the best available alternatives^37^, while a societal perspective yielded $1299 higher NMBs compared to a health system perspective^38–40^. However, NMBs were lower with a lifetime horizon compared to a limited one^41^, highlighting the importance of aligning the time horizon with the expected duration of intervention effects^42,43^. In this review, EEs of AI-based digital diagnostics often used a lifetime horizon to capture long-term benefits from a one-time diagnosis, whereas EEs of other AI-PM types typically adopted shorter time horizons (under 3 years) due to continuous use and frequent updates.

A key concern was that NMBs varied substantially depending on system-level factors after LASSO regularization, which may indicate systematic study bias. First, AI-PM interventions in high-income countries were associated with $776 higher NMBs. This may be attributable to better access to high-quality, country-specific data, more established evaluations, and greater potential to achieve cost savings through workflow optimization and automation in those high-labor-cost settings (e.g., replacing labor, streamlining processes, and reducing labor expenditures). Second, and more concerning, EEs funded by private-for-profit agencies yielded $248 higher NMBs compared to those funded by public agencies. Previous studies have noted that private funding may introduce bias through over-optimism, speculative assumptions, or selective reporting of positive outcomes^37,44,45^. These findings aligned with the above observation that key modeling choices (perspective, time horizon, comparator) were essential predictors of AI-PM’s NMBs, indicating potential risk of study manipulation to bias the cost-effectiveness results. The ECOBIAS assessment further confirmed these concerns, revealing that over one-third of included EEs exhibited a high risk of bias due to a narrow perspective, limited time horizon, and inefficient comparator (e.g., best alternatives not chosen).

All current findings suggested an urgent need to standardize methodologies for AI-PM EEs, ensuring AI-PM’s specific intervention pathways are appropriately addressed and potential sources of systematic bias are mitigated. While the Consolidated Health Economic Evaluation Reporting Standards (CHEERS) checklist has been extended to AI evaluations^46^, the CHEERS for Interventions that use AI (CHEERS-AI) checklist mainly serves to standardize the reporting process for transparency and reproducibility purposes, but lacks detailed methodological guidance. Alternatively, complex technology models such as Integrated Health Technology Assessment for Evaluating Complex Technologies (INTEGRATE-HTA)^47^ could provide a step-wise approach to evaluate AI-PM but require intensive inputs from local experts, posing challenges to resource-limited countries. As such, a universally accepted framework for conducting AI-PM EEs may be beneficial to ensure consistency and transferability across studies, improve evidence-informed and context-based decision making, and reduce the burden of resource-limited countries to conduct independent assessments.

Notably, the regulation, financing, and reimbursement of AI-PM require considerations beyond monetary value, including technical, organizational, ethical, and translational aspects^30,48^. Although AI-PM tools, particularly for digital diagnostics and risk prediction, tended to be cost-effective, gaps identified in the assessment of additional value factors, which are critical for a comprehensive understanding of the multi-dimensional implications of AI-PM, continue to pose challenges for reimbursement decisions. For instance, technical readiness, ethical and legal considerations, and patient/user experience were not covered in most current EEs of AI-PM. The inadequate evaluation and reporting of these key factors not only deviated from CHEERS-AI guideline^46^, but also introduced uncertainties surrounding real-world implications, which may translate into financial and clinical risks and thus delay the reimbursement decisions. Adaptability remains another key challenge in this review, because 31% of AI-PM EEs were classified as non-adaptable and 63% lacked clarity on adaptability. Without robust evidence that AI-PMs can function in a cost-effective manner across diverse patient cohorts, reimbursement may be restricted to limited pilot programs rather than full-scale integration into healthcare systems^49^. Therefore, increased attention should be given to these additional value factors of AI-PM in future EEs to address current gaps and enhance the real-world implementation and uptake of these promising technologies.

This study has several limitations. First, we did not perform meta-analysis or meta-regression due to extensive missing data in standard errors as well as complexity and uncertainty in its computation^28^, which may reduce the robustness of findings. Second, although the regression analyses identified key sources of value heterogeneity across studies with minimal random intercept variance, high residual variance suggested unaddressed within-study heterogeneity, likely from multiple correlated observations of sensitivity or scenario analyses, but with unmeasured factors. Moreover, AI-PMs represent a broad and heterogeneous category, with their costs and effects varying considerably across conditions and contexts. While our extensive covariate adjustment and random-effects specification adjusted for observed heterogeneity and unobserved study-level clustering, they could not capture all sources of value heterogeneity. Third, our quantitative analysis was limited to studies using disability-adjusted life-years (DALYs) or QALYs. Many CEAs were excluded due to heterogeneous outcomes measured in natural units (e.g., tooth retention years for AI-aided caries detection^50–53^), limiting our ability to fully capture the uncertainty in cost-effectiveness estimates. Fourth, the WTP threshold for NMB calculation was set at country-specific one-capita GDP, but empirical methods and criteria for defining WTP thresholds varied globally, and not all countries have established thresholds. Fifth, while NMB clearly showed value size and distinguished between cost-saving and dominated outcomes^54^, value-for-money interpretation is typically binary and threshold-based. Thus, our ICER-based findings remained a critical complement, even though they could not be directly synthesized here. Sixth, this review was focused on evidence between 2013 and 2023. Given the rapid rise in AI-based digital diagnostic evaluations but limited progress in other types, disease-specific reviews are warranted for the former in future research, while emerging applications (e.g., generative AI, modular DHTs) still lack economic evidence.

This study is the first to quantitatively summarize cost-effectiveness evidence of AI-PM, a promising subcategory of AI-based health interventions, across various contexts and conditions. To conclude, our findings support the potential of AI-PM to reduce costs and improve health outcomes. Overall, 89% of base-case ICERs were reported as dominant or cost-effective under the original analysis criteria, though high value heterogeneity and varied WTP thresholds may limit the certainty and generalizability of the results. Most economic evidence on AI-PM focused on digital diagnostics and risk prediction tools, but remained scarce for self-management and precision treatment tools. Despite a tendency towards positive value for money, significant uncertainties remained in the reimbursement and adoption of AI-PM, due to low or unclear adaptability and inadequate evaluation and reporting of additional value factors that are critical for a comprehensive understanding of AI-PM’s multi-dimensional implications (e.g., technical readiness, ethical and legal considerations, and patient/user experience). Essential predictors of NMBs, as identified by LASSO-regularized mixed-effects models, include AI-PM features (AI-PM unit costs, compliance with AI-informed decisions), modeling choices (study perspective, time horizon, comparator), and system-level factors (country-income level, funder type); the latter two suggested potential systematic and study bias, which was confirmed by the ECOBIAS assessment. These findings highlight an urgent need to standardize the conduct and reporting of AI-PM EEs.

## Methods

The review was conducted following the Centre for Reviews and Dissemination (CRD) Guidance for Undertaking Reviews in Health Care^55^ and reported in accordance with the PRISMA guidelines^56^ (Supplementary Table 5).

### Systematic literature search

We performed a systematic search of EEs on AI-PM interventions compared with non-AI interventions (e.g., usual care, conventional risk scores) that were published from 2013 to 2023. Studies published before 2013 were excluded, as the rapid evolution of AI technology would have rendered them less relevant. The intervention of interest must fall within one of the four predefined AI-PM categories: digital diagnostics, risk prediction, precision treatment, or disease self-management. Studies evaluating AI-based digital health that did not enable patient-tailored decision-making or PM tools not integrated with AI algorithms were excluded. The Embase, MEDLINE Ovid, Web of Science, the International HTA Database, and the Tufts Registry databases were searched on November 3, 2023, for relevant studies published in or translated into English. All types of original research of EEs pertaining to human subjects, including BIAs, CMAs, CEAs, and CUAs, were included.

The search strategy used a combination of terms related to “economic evaluation OR cost-effectiveness OR cost-utility OR cost-benefit OR cost minimization”, “artificial intelligence OR machine learning OR deep learning OR learning algorithm”, and “precision medicine OR personalized health”. We developed a sensitive search strategy that included both subject and free-text terms for Embase and Medline, which was then translated and tailored for each of the other databases. The detailed search strategies and results for each database can be found in Supplementary Table 6. Initial title/abstract screening and full-text review were conducted by two of the three independent investigators (Y.Z., Z.L., and T.P.) based on the inclusion and exclusion criteria, with any discrepancies resolved by discussion or, where necessary, by a third investigator until consensus was reached. The papers that met the criteria were finally verified by a fourth expert investigator (W.C.) to confirm eligibility.

A data extraction form was developed based on the key elements suggested by the CRD’s Guidance for Undertaking Reviews in Health Care^55^. Data, extracted by three independent investigators (Y.Z., Z.L., and T.P.), included study characteristics (authors, publication year, study currency, currency year, geographic region, country-income level, target population, disease domain, funder type, developmental stage at evaluation [i.e., market-access and early], and conflict of interest), methodological aspects (EE type, model type, study perspective, time horizon, discount rate, data source, original WTP threshold, and comparators), and intervention features (type of AI-PM tools [i.e., digital diagnostics, risk prediction, precision treatment, and disease self-management], type of digital/electronic products [i.e., software, interactive application, interconnected device, and standalone algorithm], unit costs of the AI-PM intervention, AI performance, adherence to AI technology, and compliance with AI-informed decisions). Additionally, we extracted the cost-effectiveness profiles, including Δcosts, ΔQALYs, and ICERs for the intervention arm relative to the comparator arm, from both base-case and sensitivity analyses. The ICER is a ratio measure quantifying the additional cost required to gain one unit of health benefit, with a lower value indicating better value for money. An intervention is considered cost-effective if the calculated ICER falls below the predefined WTP threshold. If studies included multiple interventions, comparators, or disease conditions, all two-way comparisons between AI-PM and non-AI interventions were recorded. Risk of bias was assessed using the ECOBIAS checklist^57^, which was only applicable to model-based EEs. Furthermore, we performed additional value assessment to capture the remaining enablers and barriers to AI-PM adoption in clinical practice, including patient/user experience, use case validity, technical readiness, ethical and legal considerations, and adaptability.

### Statistical analysis

To enable cross-comparisons by disease contexts and study conditions, we summarized the value for money of AI-PM based on cost-utility analyses. Cost-utility analysis assesses the health gains in standardized outcome measures, such as DALYs or QALYs^58^. In addition to the extracted ICER^59^, we calculated the NMB of each comparison. A positive NMB indicates the intervention is cost-effective (or dominant) over the comparator, whereas a negative NMB indicates that the intervention is not cost-effective. NMB was calculated as per-person values using the following formula:

$$(Incremental\,effectiveness\times willingness-to-pay\,threshold)-Incremental\,cost$$

According to WHO recommendations, the one-time per-capita GDP of the currency year was used as the WTP threshold to place NMB within the economic context of each country, enabling global comparison^60^. For standardization, all cost parameters were inflated to the 2023 currency of the study country using the country-specific consumer price index, and then converted to 2023 United States dollars using the exchange rate from World Bank^61,62^.

First, descriptive analyses were performed to compare the mean, SD, median, IQR (25th–75th percentiles), and minimum and maximum values across intervention types and developmental stages. Using box plots, we summarized the distributions of Δcosts, ΔQALYs, and NMBs, both overall and by these subgroups. The Mann–Whitney U test was used for comparison between subgroups, where a two-sided *p* value < 0.05 was considered statistically significant. Of note, we did not perform a formal meta-analysis nor meta-regression despite the availability of suitable methods (e.g., Comparative efficiency research^63^), because a key component, the standard error of these endpoints, is rarely reported. Estimating it would require substantial approximation and Monte Carlo simulation, which may hardly address the high heterogeneity of cost-effectiveness values but rather introduce additional uncertainty and reduce the robustness of study findings.

Next, to better reflect the dual nature of decision-making for value-for-money, we further compared ICERs in a common cost-effectiveness plane to assess the trade-offs between costs and effects across study perspectives and comparators, with country settings, AI-PM intervention types, and conclusions (dominant, cost-effective, etc.) labeled accordingly.

In the following, to investigate the heterogeneity in the cost-effectiveness profiles, we conducted a series of regression analyses stepwise. First, to capture heterogeneity across multiple economic dimensions, we used Δcosts, ΔQALYs, and NMBs as the summary measure for regression analyses^45,64^. Each step involved three separate models with Δcosts, ΔQALYs, or NMBs as the dependent variables. NMB was chosen over ICER for regression analyses because the former follows the central limit theorem and tends to be normally distributed, whereas the latter as a ratio is typically skewed and reflects policy duality, thus less suitable for associational study. In addition, one published review^54^ that used ICERs as the endpoint for regression analyses excluded dominated and cost-saving results, which would be impractical and infeasible for synthesizing evidence on emerging interventions with limited available data. Second, to capture unobserved heterogeneity, linear mixed models with a Gaussian distribution were used, with random intercepts for each study to account for between-study variability. Third, for all three models, we included a broad set of 17 potential risk factors to address observed heterogeneity in multi-dimensional economic endpoints, covering study contexts (disease domain by international classification of diseases, 11th revision [ICD-11]^65^, country income level^66^, WHO geographical region^67^, target population age, target population sex, funder type, and developmental stage), intervention features (type of intervention, type of digital/electronic products, AI-PM unit cost, integration of AI performance, integration of adherence to AI technology, and integration of compliance with AI-informed decisions), and study methods (choice of comparators, study perspective, time horizon, and usage of EE guidance/reference case). In step one, univariate mixed-effects regressions were employed to assess how each of the 17 candidate predictors is individually associated with the reported cost-effectiveness of AI-PM. In the following step, we applied the penalized Lasso regression with ten-fold cross-validation, a well-established machine learning approach, to identify essential predictors of value heterogeneity^68^, which minimized overfitting and optimized model parsimony. The selected covariates were then analyzed using multivariate mixed-effects regressions to quantify their associations with Δcosts, ΔQALYs, and NMBs, respectively.

### Reporting summary

Further information on research design is available in the Nature Research Reporting Summary linked to this article.

## Supplementary information

Supplementary Material

Reporting Summary

## Data Availability

Data collected and used in this systematic review and regression analysis can be requested from the corresponding author.
