# Supplementary material for: The value for money of artificial intelligence-empowered precision medicine: a systematic review and regression analysis
Source: NPJ Digit Med. 2025 Dec 24;9:78. doi: 10.1038/s41746-025-02259-w (PMC12848305; doi:10.1038/s41746-025-02259-w)
Supplement: Supplementary file 1 — Supplementary Material [file 41746_2025_2259_MOESM1_ESM.pdf]

Supplemental Information

The value for money of artificial intelligence-empowered precision medicine: a systematic review and regression analysis

Supplementary Table 1. References of included studies

Supplementary Figure 1. Time trend of included EEs based on types of AI-PM tools

Supplementary Table 2. Study, methodological, and intervention characteristics of the included studies overall and by AI-PM types

Supplementary Table 3. Details of the top five AI-PM interventions in terms of NMBs, △costs, and △QALYs

Supplementary Table 4. The univariate mixed-effects regression results

Supplementary Figure 2. Cost-effectiveness profiles of AI-PM tools plotted against unit cost

Supplementary Table 5. Preferred Reporting Items for Systematic Reviews and Meta-analyses (PRISMA) checklist

Supplementary Table 6. Search strategy and results

Supplementary Table 1. References of included studies

1. Adams SJ, Mondal P, Penz E, Tyan CC, Lim H, Babyn P. Development and Cost Analysis of a Lung Nodule Management Strategy Combining Artificial Intelligence and Lung-RADS for Baseline Lung Cancer Screening. *J Am Coll Radiol*. May 2021;18(5):741-751. doi:10.1016/j.jacr.2020.11.014
2. Areia M, Mori Y, Correale L, et al. Cost-effectiveness of artificial intelligence for screening colonoscopy: a modelling study. *Lancet Digit Health*. Jun 2022;4(6):e436-e444. doi:10.1016/S2589-7500(22)00042-5
3. Barkun AN, von Renteln D, Sadri H. Cost-effectiveness of Artificial Intelligence-Aided Colonoscopy for Adenoma Detection in Colon Cancer Screening. *J Can Assoc Gastroenterol*. Jun 2023;6(3):97-105. doi:10.1093/jcag/gwad014
4. Bashir S, Kik SV, Ruhwald M, et al. Economic analysis of different throughput scenarios and implementation strategies of computer-aided detection software as a screening and triage test for pulmonary TB. *PLoS One*. 2022;17(12):e0277393. doi:10.1371/journal.pone.0277393
5. Chawla H, Uhr JH, Williams JS, Reinoso MA, Weiss JS. Economic Evaluation of Artificial Intelligence Systems Versus Manual Screening for Diabetic Retinopathy in the United States. *Ophthalmic Surg Lasers Imaging Retina*. May 2023;54(5):272-280. doi:10.3928/23258160-20230406-01
6. Datar M, Burchenal W, Donovan MJ, Coca SG, Wang E, Goss TF. Payer budget impact of an artificial intelligence in vitro diagnostic to modify diabetic kidney disease progression. *J Med Econ*. Jan-Dec 2021;24(1):972-982. doi:10.1080/13696998.2021.1960714
7. de Vos J, Visser LA, de Beer AA, et al. The Potential Cost-Effectiveness of a Machine Learning Tool That Can Prevent Untimely Intensive Care Unit Discharge. *Value Health*. Mar 2022;25(3):359-367. doi:10.1016/j.jval.2021.06.018
8. Ericson O, Hjelmgren J, Sjovall F, Soderberg J, Persson I. The Potential Cost and Cost-Effectiveness Impact of Using a Machine Learning Algorithm for Early Detection of Sepsis in Intensive Care Units in Sweden. *J Health Econ Outcomes Res*. 2022;9(1):101-110. doi:10.36469/jheor.2022.33951
9. Esposito G, Ernst B, Henket M, et al. AI-Based Chest CT Analysis for Rapid COVID-19 Diagnosis and Prognosis: A Practical Tool to Flag High-Risk Patients and Lower Healthcare Costs. *Diagnostics (Basel)*. Jul 1 2022;12(7)doi:10.3390/diagnostics12071608
10. Fuller SD, Hu J, Liu JC, et al. Five-Year Cost-Effectiveness Modeling of Primary Care-Based, Nonmydriatic Automated Retinal Image Analysis Screening Among Low-Income Patients With Diabetes. *J Diabetes Sci Technol*. Mar 2022;16(2):415-427. doi:10.1177/1932296820967011
11. Fusfeld L, Menon S, Gupta G, Lawrence C, Masud SF, Goss TF. US payer budget impact of a microarray assay with machine learning to evaluate kidney transplant rejection in for-cause biopsies. *J Med Econ*. Jan-Dec 2022;25(1):515-523. doi:10.1080/13696998.2022.2059221
12. Gomez Rossi J, Rojas-Perilla N, Krois J, Schwendicke F. Cost-effectiveness of Artificial Intelligence as a Decision-Support System Applied to the Detection and Grading of Melanoma, Dental Caries, and Diabetic Retinopathy. *JAMA Netw Open*. Mar 1 2022;5(3):e220269. doi:10.1001/jamanetworkopen.2022.0269
13. Hassan C, Povero M, Pradelli L, Spadaccini M, Repici A. Cost-utility analysis of real-time artificial intelligence-assisted colonoscopy in Italy. *Endosc Int Open*. Nov 2023;11(11):E1046-E1055. doi:10.1055/a-2136-3428
14. Hill NR, Groves L, Dickerson C, et al. Identification of undiagnosed atrial fibrillation using a machine learning risk prediction algorithm and diagnostic testing (PULsE-AI) in primary care: cost-effectiveness of a screening strategy evaluated in a randomized controlled trial in England. *J Med Econ*. Jan-Dec 2022;25(1):974-983. doi:10.1080/13696998.2022.2102355
15. Hill NR, Sandler B, Mokgokong R, et al. Cost-effectiveness of targeted screening for the identification of patients with atrial fibrillation: evaluation of a machine learning risk prediction algorithm. *J Med Econ*. Apr 2020;23(4):386-393. doi:10.1080/13696998.2019.1706543
16. Huang XM, Yang BF, Zheng WL, et al. Cost-effectiveness of artificial intelligence screening for diabetic retinopathy in rural China. *BMC Health Serv Res*. Feb 25 2022;22(1):260. doi:10.1186/s12913-022-07655-6

17. Kacew AJ, Strohbehn GW, Saulsberry L, et al. Artificial Intelligence Can Cut Costs While Maintaining Accuracy in Colorectal Cancer Genotyping. *Front Oncol*. 2021;11:630953. doi:10.3389/fonc.2021.630953
18. Lin S, Ma Y, Xu Y, et al. Artificial Intelligence in Community-Based Diabetic Retinopathy Telemedicine Screening in Urban China: Cost-effectiveness and Cost-Utility Analyses With Real-world Data. *JMIR Public Health Surveill*. Feb 23 2023;9:e41624. doi:10.2196/41624
19. Lin Y, Huang S, Simon GE, Liu S. Cost-effectiveness analysis of prognostic-based depression monitoring. *IISE Transactions on Healthcare Systems Engineering*. 2019/01/02 2019;9(1):41-54. doi:10.1080/24725579.2019.1567627
20. Liu H, Li R, Zhang Y, et al. Economic evaluation of combined population-based screening for multiple blindness-causing eye diseases in China: a cost-effectiveness analysis. *Lancet Glob Health*. Mar 2023;11(3):e456-e465. doi:10.1016/S2214-109X(22)00554-X
21. MacPherson P, Webb EL, Kamchedzera W, et al. Computer-aided X-ray screening for tuberculosis and HIV testing among adults with cough in Malawi (the PROSPECT study): A randomised trial and cost-effectiveness analysis. *PLoS Med*. Sep 2021;18(9):e1003752. doi:10.1371/journal.pmed.1003752
22. Mallow PJ, Belk KW. Cost-utility analysis of single nucleotide polymorphism panel-based machine learning algorithm to predict risk of opioid use disorder. *J Comp Eff Res*. Dec 2021;10(18):1349-1361. doi:10.2217/cer-2021-0115
23. Mervin MC, Moyle W, Jones C, et al. The Cost-Effectiveness of Using PARO, a Therapeutic Robotic Seal, to Reduce Agitation and Medication Use in Dementia: Findings from a Cluster-Randomized Controlled Trial. *J Am Med Dir Assoc*. Jul 2018;19(7):619-622 e1. doi:10.1016/j.jamda.2017.10.008
24. Mital S, Nguyen HV. Cost-effectiveness of using artificial intelligence versus polygenic risk score to guide breast cancer screening. *BMC Cancer*. May 6 2022;22(1):501. doi:10.1186/s12885-022-09613-1
25. Morrison SL, Dukhovny D, Chan RVP, Chiang MF, Campbell JP. Cost-effectiveness of Artificial Intelligence-Based Retinopathy of Prematurity Screening. *JAMA Ophthalmol*. Apr 1 2022;140(4):401-409. doi:10.1001/jamaophthalmol.2022.0223
26. Nsengiyumva NP, Hussain H, Oxlade O, et al. Triage of Persons With Tuberculosis Symptoms Using Artificial Intelligence-Based Chest Radiograph Interpretation: A Cost-Effectiveness Analysis. *Open Forum Infect Dis*. Dec 2021;8(12):ofab567. doi:10.1093/ofid/ofab567
27. Pickhardt PJ, Correale L, Hassan C. AI-based opportunistic CT screening of incidental cardiovascular disease, osteoporosis, and sarcopenia: cost-effectiveness analysis. *Abdom Radiol (NY)*. Mar 2023;48(3):1181-1198. doi:10.1007/s00261-023-03800-9
28. Salcedo J, Rosales M, Kim JS, Nuno D, Suen SC, Chang AH. Cost-effectiveness of artificial intelligence monitoring for active tuberculosis treatment: A modeling study. *PLoS One*. 2021;16(7):e0254950. doi:10.1371/journal.pone.0254950
29. Schwendicke F, Cejudo Grano de Oro J, Garcia Cantu A, Meyer-Lueckel H, Chaurasia A, Krois J. Artificial Intelligence for Caries Detection: Value of Data and Information. *J Dent Res*. Oct 2022;101(11):1350-1356. doi:10.1177/00220345221113756
30. Schwendicke F, Mertens S, Cantu AG, Chaurasia A, Meyer-Lueckel H, Krois J. Cost-effectiveness of AI for caries detection: randomized trial. *J Dent*. Apr 2022;119:104080. doi:10.1016/j.jdent.2022.104080
31. Schwendicke F, Rossi JG, Gostemeyer G, et al. Cost-effectiveness of Artificial Intelligence for Proximal Caries Detection. *J Dent Res*. Apr 2021;100(4):369-376. doi:10.1177/0022034520972335
32. Sharma V, Kulkarni V, Joon T, et al. Predicting falls-related admissions in older adults in Alberta, Canada: a machine-learning falls prevention tool developed using population administrative health data. *BMJ Open*. Aug 22 2023;13(8):e071321. doi:10.1136/bmjopen-2022-071321
33. Shen M, Zou Z, Bao H, et al. Cost-effectiveness of artificial intelligence-assisted liquid-based cytology testing for cervical cancer screening in China. *Lancet Reg Health West Pac*. May 2023;34:100726. doi:10.1016/j.lanwpc.2023.100726
34. Shung DL, Lin JK, Laine L. Achieving Value by Risk Stratification With Machine Learning Model or Clinical Risk Score in Acute Upper Gastrointestinal Bleeding: A Cost Minimization Analysis. *Am J Gastroenterol*. Feb 1 2024;119(2):371-373. doi:10.14309/ajg.0000000000002520

35. Skarping I, Nilsson K, Dihge L, et al. The implementation of a noninvasive lymph node staging (NILS) preoperative prediction model is cost effective in primary breast cancer. *Breast Cancer Res Treat*. Aug 2022;194(3):577-586. doi:10.1007/s10549-022-06636-x
36. Srisubat A, Kittrongsiri K, Sangroongruangsri S, et al. Cost-Utility Analysis of Deep Learning and Trained Human Graders for Diabetic Retinopathy Screening in a Nationwide Program. *Ophthalmol Ther*. Apr 2023;12(2):1339-1357. doi:10.1007/s40123-023-00688-y
37. Tariq A, Lancaster L, Elugunti P, et al. Graph convolutional network-based fusion model to predict risk of hospital acquired infections. *J Am Med Inform Assoc*. May 19 2023;30(6):1056-1067. doi:10.1093/jamia/ocad045
38. Thiruvengadam NR, Cote GA, Gupta S, et al. An Evaluation of Critical Factors for the Cost-Effectiveness of Real-Time Computer-Aided Detection: Sensitivity and Threshold Analyses Using a Microsimulation Model. *Gastroenterology*. May 2023;164(6):906-920. doi:10.1053/j.gastro.2023.01.027
39. Tseng AS, Thao V, Borah BJ, et al. Cost Effectiveness of an Electrocardiographic Deep Learning Algorithm to Detect Asymptomatic Left Ventricular Dysfunction. *Mayo Clin Proc*. Jul 2021;96(7):1835-1844. doi:10.1016/j.mayocp.2020.11.032
40. Turino C, Benitez ID, Rafael-Palou X, et al. Management and Treatment of Patients With Obstructive Sleep Apnea Using an Intelligent Monitoring System Based on Machine Learning Aiming to Improve Continuous Positive Airway Pressure Treatment Compliance: Randomized Controlled Trial. *J Med Internet Res*. Oct 18 2021;23(10):e24072. doi:10.2196/24072
41. van Leeuwen KG, Meijer FJA, Schalekamp S, et al. Cost-effectiveness of artificial intelligence aided vessel occlusion detection in acute stroke: an early health technology assessment. *Insights Imaging*. Sep 25 2021;12(1):133. doi:10.1186/s13244-021-01077-4
42. Wang HY, Hung CC, Chen CH, et al. Increase Trichomonas vaginalis detection based on urine routine analysis through a machine learning approach. *Sci Rep*. Aug 19 2019;9(1):11074. doi:10.1038/s41598-019-47361-8
43. Wolf RM, Channa R, Abramoff MD, Lehmann HP. Cost-effectiveness of Autonomous Point-of-Care Diabetic Retinopathy Screening for Pediatric Patients With Diabetes. *JAMA Ophthalmol*. Oct 1 2020;138(10):1063-1069. doi:10.1001/jamaophthalmol.2020.3190
44. Xiao X, Xue L, Ye L, Li H, He Y. Health care cost and benefits of artificial intelligence-assisted population-based glaucoma screening for the elderly in remote areas of China: a cost-offset analysis. *BMC Public Health*. Jun 4 2021;21(1):1065. doi:10.1186/s12889-021-11097-w
45. Xie Y, Nguyen Q, Bellemo V, et al. Cost-Effectiveness Analysis of an Artificial Intelligence-Assisted Deep Learning System Implemented in the National Tele-Medicine Diabetic Retinopathy Screening in Singapore. *Investigative Ophthalmology & Visual Science*. 2019;60(9):5471-5471.
46. Xie Y, Nguyen QD, Hamzah H, et al. Artificial intelligence for teleophthalmology-based diabetic retinopathy screening in a national programme: an economic analysis modelling study. *Lancet Digit Health*. May 2020;2(5):e240-e249. doi:10.1016/S2589-7500(20)30060-1
47. Yonazu S, Ozawa T, Nakanishi T, et al. Cost-effectiveness analysis of the artificial intelligence diagnosis support system for early gastric cancers. *DEN Open*. Apr 2024;4(1):e289. doi:10.1002/deo2.289
48. Ziegelmayer S, Graf M, Makowski M, Gawlitza J, Gassert F. Cost-Effectiveness of Artificial Intelligence Support in Computed Tomography-Based Lung Cancer Screening. *Cancers (Basel)*. Mar 29 2022;14(7)doi:10.3390/cancers14071729

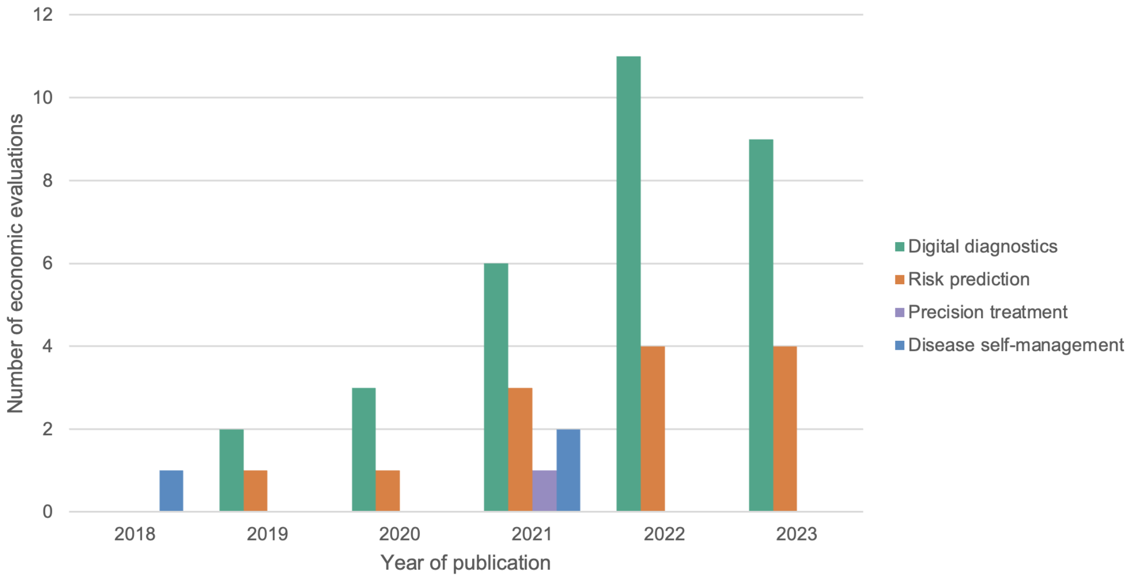

Supplementary Figure 1. Time trend of included EEs based on types of AI-PM tools (N = 48). *AI* artificial intelligence, *EE* economic evaluation, *PM* precision medicine. The figure shows the annual number of published economic evaluations of artificial intelligence-empowered precision medicine (AI-PM) from 2018 to November 2023, stratified by AI-PM types, including digital diagnostics, risk prediction, precision treatment, and disease self-management.

Supplementary Table 2. Study, methodological, and intervention characteristics of the included studies overall and by AI-PM types

Supplementary Table 2.1 Study characteristics of the included studies overall and by AI-PM types

Category	Subcategory	N (%)				
		Overall (N=50)[a]	Digital diagnostics (N=33)	Risk prediction (N=13)	Precision treatment (N=1)	Disease self-management (N=3)
Country/region of analysis	Canada	3 (6.0)	1 (3.0)	2 (15.4)	0 (0)	0 (0)
	China	5 (10.0)	5 (15.2)	0 (0)	0 (0)	0 (0)
	Germany	4 (8.0)	4 (12.1)	0 (0)	0 (0)	0 (0)
	Pakistan	2 (4.0)	2 (6.1)	0 (0)	0 (0)	0 (0)
	Singapore	2 (4.0)	2 (6.1)	0 (0)	0 (0)	0 (0)
	Sweden	2 (4.0)	1 (3.0)	1 (7.7)	0 (0)	0 (0)
	United Kingdom	3 (6.0)	1 (3.0)	2 (15.4)	0 (0)	0 (0)
	United States of America	19 (38.0)	10 (30.3)	7 (53.8)	1 (100)	1 (33.3)
	Others[b]	10 (20.0)	7 (21.2)	1 (7.7)	0 (0)	2 (66.7)
Target population—age	Pediatric	4 (8.0)	4 (12.1)	0 (0)	0 (0)	0 (0)
	Adult	26 (52.0)	17 (51.5)	8 (61.5)	0 (0)	1 (33.3)
	Senior	5 (10.0)	3 (9.1)	1 (7.7)	0 (0)	1 (33.3)
	All ages	6 (12.0)	3 (9.1)	2 (15.4)	1 (100)	0 (0)
	Not specified	9 (18.0)	6 (18.2)	2 (15.4)	0 (0)	1 (33.3)
Target population—sex	All male	0 (0)	0 (0)	0 (0)	0 (0)	0 (0)
	All female	3 (6.0)	2 (6.1)	1 (7.7)	0 (0)	0 (0)
	Gender/sex-neutral	47 (94.0)	31 (93.9)	12 (92.3)	1 (100)	3 (100.0)
Disease domain (ICD-11 category)	Certain infectious or parasitic diseases	7 (14.0)	5 (15.2)	1 (7.7)	0 (0)	1 (33.3)
	Diseases of the circulatory system	3 (6.0)	1 (3.0)	2 (15.4)	0 (0)	0 (0)
	Diseases of the digestive system	5 (10.0)	4 (12.1)	1 (7.7)	0 (0)	0 (0)
	Diseases of the nervous system	2 (4.0)	1 (3.0)	0 (0)	0 (0)	1 (33.3)
	Diseases of the visual system	12 (24.0)	12 (36.4)	0 (0)	0 (0)	0 (0)
	Injury, poisoning or external causes	3 (6.0)	1 (3.0)	2 (15.4)	0 (0)	0 (0)
	Mental, behavioral or neurodevelopmental disorders	3 (6.0)	0 (0)	2 (15.4)	0 (0)	1 (33.3)
	Neoplasms	12 (24.0)	9 (27.3)	2 (15.4)	1 (100)	0 (0)
	Others[c]	3 (6.0)	0 (0)	3 (23.1)	0 (0)	0 (0)
Funder type	Public	16 (32.0)	12 (36.4)	2 (15.4)	0 (0)	2 (66.7)
	Private – not for profit	3 (6.0)	2 (6.1)	1 (7.7)	0 (0)	0 (0)
	Private – for profit	9 (18.0)	3 (9.1)	6 (46.2)	0 (0)	0 (0)
	Mixed funding source	6 (12.0)	4 (12.1)	0 (0)	1 (100)	1 (33.3)
	No funding source	8 (16.0)	6 (18.2)	2 (15.4)	0 (0)	0 (0)
	Not stated	8 (16.0)	6 (18.2)	2 (15.4)	0 (0)	0 (0)
Developmental stage	Early stage	10 (20.0)	5 (15.2)	5 (38.4)	0 (0)	0 (0)
	Market-access stage	40 (80.0)	28 (84.8)	8 (61.5)	1 (100)	3 (100)

[a] N = 50 as one study analyzed 3 AI-PM interventions, which was accounted for thrice, resulting in a total of 50 datasets in base-case descriptive analysis.
[b] The subcategory "Others" included 1 study from Australia, 1 study from Brazil, 1 study from Belgium, 1 study from Italy, 1 study from Japan, 1 study from Malawi, 1 study from Spain, 1 study from Taiwan, 1 study from Thailand, and 1 study from the Netherlands.
[c] The sub-category "Others" included 1 study under diseases of the genitourinary system, 1 study under diseases of the musculoskeletal system or connective tissue, and 1 study under not specified.

Supplementary Table 2.2 Methodological characteristics of the included studies overall and by AI-PM types

Category	Sub-category	N (%)				
		Overall (N=50)[a]	Digital diagnostics (N=33)	Risk prediction (N=13)	Precision treatment (N=1)	Disease self-management (N=3)
Economic evaluation type	Budget impact analysis	6 (12.0)	2 (6.1)	3 (23.1)	1 (100)	0 (0)
	Cost minimization analysis	3 (6.0)	2 (6.1)	1 (7.7)	0 (0)	0 (0)
	Cost effectiveness analysis	10 (20.0)	7 (21.2)	1 (7.7)	0 (0)	2 (66.7)
	Cost utility analysis	28 (56.0)	19 (57.6)	8 (61.5)	0 (0)	1 (33.3)
	Cost effectiveness and cost utility analysis	3 (6.0)	3 (9.1)	0 (0)	0 (0)	0 (0)
Model type	Modeling—Markov	15 (30.0)	11 (33.3)	3 (23.1)	0 (0)	1 (33.3)
	Modeling—decision tree	11 (22.0)	7 (21.2)	3 (23.1)	1 (100)	0 (0)
	Hybrid model	12 (24.0)	8 (24.2)	4 (30.8)	0 (0)	0 (0)
	Patient-level simulation	4 (8.0)	4 (12.1)	0 (0)	0 (0)	0 (0)
	Other type of analysis (non-model)	8 (16.0)	3 (9.1)	3 (23.1)	0 (0)	2 (66.7)
Time horizon	Short term (≤3 years)	14 (28.0)	5 (15.2)	5 (38.5)	1 (100)	3 (100)
	Intermediate term (3-10 years)	10 (20.0)	6 (18.2)	4 (30.8)	0 (0)	0 (0)
	Long term (10-30 years)	2 (4.0)	2 (6.1)	0 (0)	0 (0)	0 (0)
	Lifetime (>30 years)	23 (46.0)	19 (57.6)	4 (30.8)	0 (0)	0 (0)
	Not applicable	1 (2.0)	1 (3.0)	0 (0)	0 (0)	0 (0)
Study perspective	Societal	9 (18.0)	7 (21.2)	1 (7.7)	0 (0)	1 (33.3)
	Health system	38 (76.0)	25 (75.8)	10 (76.9)	1 (100)	2 (66.7)
	Patient	1 (2.0)	1 (3.0)	0 (0)	0 (0)	0 (0)
	Not specified	2 (4.0)	0 (0)	2 (15.4)	0 (0)	0 (0)

[a] N = 50 as one study analyzed 3 AI-PM interventions, which was accounted for thrice, resulting in a total of 50 datasets in base-case descriptive analysis.

Supplementary Table 2.3 Intervention characteristics of the included studies overall and by AI-PM types

Category	Sub-category	N (%)				
		Overall (N=50)[a]	Digital diagnostics (N=33)	Risk prediction (N=13)	Precision treatment (N=1)	Disease self-management (N=3)
Type of digital/electronic product	Software	34 (68.0)	28 (84.8)	6 (46.2)	0 (0)	2 (66.7)
	Interactive application	3 (6.0)	0 (0)	1 (7.7)	0 (0)	0 (0)
	Device	1 (2.0)	0 (0)	0 (0)	0 (0)	1 (33.3)
	Standalone algorithm	12 (24.0)	5 (15.2)	6 (46.2)	1 (100)	0 (0)
Integration of AI performance rate	Yes	40 (80.0)	29 (87.9)	10 (76.9)	1 (100)	0 (0)
	No	10 (20.0)	4 (12.1)	3 (23.1)	0 (0)	3 (100)
Integration of adherence to AI technology	Yes	3 (6.0)	3 (9.1)	0 (0)	0 (0)	0 (0)
	No	47 (94.0)	30 (90.9)	13 (100)	1 (100)	3 (100)
Integration of compliance with AI-informed decisions	Yes	6 (12.0)	4 (12.1)	2 (15.4)	0 (0)	0 (0)
	No	44 (88.0)	29 (87.9)	11 (84.6)	1 (100)	3 (100)
Direct reporting of AI-PM costs	Yes	43 (86.0)	30 (90.9)	9 (69.2)	1 (100)	3 (100)
	No	7 (14.0)	3 (9.1)	4 (30.8)	0 (0)	0 (0)

Category	Sub-category	N (%)				
		Overall (N=50)[a]	Digital diagnostics (N=33)	Risk prediction (N=13)	Precision treatment (N=1)	Disease self-management (N=3)
AI-PM unit cost, (median [interquartile range])		$10.20 ($1.12 to $51.76)	$9.91 ($1.82 to $37.03)	$10 ($0 to $131.90)	$7.15 (-)	$64.43 ($52.41 to $122.11)
Provided detailed specifications of AI-PM costs	Yes	16 (32.0)	12 (36.4)	1 (7.7)	0 (0)	3 (100)
	No	34 (68.0)	21 (63.6)	12 (92.3)	1 (100)	0 (0)

[a] N = 50 as one study analyzed 3 AI-PM interventions, which was accounted for thrice, resulting in a total of 50 datasets in base-case descriptive analysis.

Supplementary Table 3. Details of the top five AI-PM interventions in terms of NMBs, Δcosts, and ΔQALYs

Supplementary Table 3.1 Details of top five AI-PM interventions with highest NMBs

NMB[a]	Intervention	Comparator	Country	Disease Domain	Intervention type	Product type	EE stage	Funder type
10669.11[1]	AI software tools to guide endoscopists in identifying polyps during colonoscopy screening by real-time pattern recognition	No colonoscopy screening	United States of America	Colorectal cancer	Digital diagnostics	Software	Market-access	Public
4503.74[2]	Use of machine learning algorithm to identify patients with a higher risk of opioid use disorder before surgery to guide analgesia prescription	Standard of care without machine learning algorithm applied	United States of America	Opioid use disorder	Risk prediction	Pure algorithm	Market-access	Private - for-profit
3967.76[3]	AI platform that allows for automated treatment monitoring for patients undergoing tuberculosis treatment	Traditional in-person direct observed therapy for tuberculosis	United States of America	Tuberculosis	Disease self-management	Interactive app	Market-access	Public
3446.57[4]	Risk-stratified mammography screening for breast cancer based on risk prediction using AI-based discriminative image pattern recognition from full-field mammograms and traditional risk factors	Annual mammography screening without AI-based risk stratification	United States of America	Breast cancer	Risk prediction	Software	Market-access	No funding
2745.34[5]	AI-assisted deep learning system screening model for retinal images for detection of diabetic retinopathy	Manual screening for diabetic retinopathy by ophthalmologist	Singapore	Diabetic retinopathy	Digital diagnostics	Software	Market-access	Not stated

AI artificial intelligence, *EE* economic evaluation, *NMB* net monetary benefit.

[a] All currency is presented in 2023 United States dollars.

Supplementary Table 3.2 Details of top five AI-PM interventions with lowest Δcosts

Δcosts[a]	Intervention	Comparator	Country	Disease Domain	Intervention type	Product type	EE stage	Funder type
−3201.33[2]	Use of machine learning algorithm to identify patients with a higher risk of opioid use disorder before surgery to guide analgesia prescription	Standard of care without machine learning algorithm applied	United States of America	Opioid use disorder	Risk prediction	Standalone algorithm	Market-access	Private - for-profit
−2769.60[3]	AI platform that allows for automated treatment monitoring for patients undergoing tuberculosis treatment	Traditional in-person direct observed therapy for tuberculosis	United States of America	Tuberculosis	Disease self-management	Interactive app	Market-access	Public
−2104.21[4]	Risk-stratified mammography screening for breast cancer based on risk prediction using AI-based discriminative image pattern recognition from full-field mammograms and traditional risk factors	Annual mammography screening without AI-based risk stratification	United States of America	Breast cancer	Risk prediction	Software	Market-access	No funding
−579[6]	AI-based automated opportunistic measures of CV risk, osteoporosis, and sarcopenia from CT scans to determine therapeutic interventions	Universal statin therapy for CV prevention regardless of CT findings	United States of America	CV disease, osteoporosis, and sarcopenia	Risk prediction	Software	Market-access	Private - for-profit
−536.96[7]	An AI network model to predict pathological nodal status in patients with clinically node-negative breast cancer, where SLNB will only be performed after a positive prediction result	SLNB for all patients with clinically node-negative breast cancer	Sweden	Breast cancer	Digital diagnostics	Standalone algorithm	Early	Public

AI artificial intelligence, *CT* computed tomography, *CV* cardiovascular, *EE* economic evaluation, *SLNB* sentinel lymph node biopsy.

[a] All currency is presented in 2023 United States dollars.

Supplementary Table 3.3 Details of top five AI-PM interventions with highest ΔQALYs

ΔQALYs	Intervention	Comparator	Country	Disease Domain	Intervention type	Product type	EE stage	Funder type
0.170[8]	AI telemedicine combined screening for multiple eye diseases	No screening (Opportunistic case finding)	China	Multiple major blindness-causing eye diseases, including age-related macular degeneration, glaucoma, diabetic retinopathy, cataracts, and pathological myopia	Digital diagnostics	Software	Market-access	Public
0.160[9]	AI-based software to grade fundus images in diabetic retinopathy screening	No screening	China	Diabetic retinopathy	Digital diagnostics	Software	Market-access	Public
0.149[1]	AI software tools to guide endoscopists in identifying polyps during colonoscopy screening by real-time pattern recognition	No colonoscopy screening	United States of America	Colorectal cancer	Digital diagnostics	Software	Market-access	Public
0.042[10]	Machine learning algorithm for forecasting the onset of sepsis in intensive care units	Sepsis diagnosis in accordance with clinical practice (Sepsis-3 criteria)	Sweden	Sepsis	Risk prediction	Software	Market-access	Private - for-profit
0.041[4]	Risk-stratified mammography screening for breast cancer based on risk prediction using AI-based discriminative image pattern recognition from full-field mammograms and traditional risk factors	No screening at any age regardless of risk level	United States of America	Breast cancer	Risk prediction	Software	Market-access	No funding

AI artificial intelligence, *EE* economic evaluation, *QALY* quality-adjusted life-years.

Reference

1. Areia M, Mori Y, Correale L, et al. Cost-effectiveness of artificial intelligence for screening colonoscopy: a modelling study. *Lancet Digit Health*. Jun 2022;4(6):e436-e444. doi:10.1016/S2589-7500(22)00042-5
2. Mallow PJ, Belk KW. Cost-utility analysis of single nucleotide polymorphism panel-based machine learning algorithm to predict risk of opioid use disorder. *J Comp Eff Res*. Dec 2021;10(18):1349-1361. doi:10.2217/cer-2021-0115
3. Salcedo J, Rosales M, Kim JS, Nuno D, Suen SC, Chang AH. Cost-effectiveness of artificial intelligence monitoring for active tuberculosis treatment: A modeling study. *PLoS One*. 2021;16(7):e0254950. doi:10.1371/journal.pone.0254950
4. Mital S, Nguyen HV. Cost-effectiveness of using artificial intelligence versus polygenic risk score to guide breast cancer screening. *BMC Cancer*. May 6 2022;22(1):501. doi:10.1186/s12885-022-09613-1
5. Xie Y, Nguyen Q, Bellemo V, et al. Cost-effectiveness analysis of an artificial intelligence-assisted deep learning system implemented in the national tele-medicine diabetic retinopathy screening in Singapore. *Investigative Ophthalmology & Visual Science*. 2019;60(9):5471-5471.
6. Pickhardt PJ, Correale L, Hassan C. AI-based opportunistic CT screening of incidental cardiovascular disease, osteoporosis, and sarcopenia: cost-effectiveness analysis. *Abdom Radiol (NY)*. Mar 2023;48(3):1181-1198. doi:10.1007/s00261-023-03800-9
7. Skarping I, Nilsson K, Dihge L, et al. The implementation of a noninvasive lymph node staging (NILS) preoperative prediction model is cost effective in primary breast cancer. *Breast Cancer Res Treat*. Aug 2022;194(3):577-586. doi:10.1007/s10549-022-06636-x
8. Liu H, Li R, Zhang Y, et al. Economic evaluation of combined population-based screening for multiple blindness-causing eye diseases in China: a cost-effectiveness analysis. *Lancet Glob Health*. Mar 2023;11(3):e456-e465. doi:10.1016/S2214-109X(22)00554-X
9. Huang XM, Yang BF, Zheng WL, et al. Cost-effectiveness of artificial intelligence screening for diabetic retinopathy in rural China. *BMC Health Serv Res*. Feb 25 2022;22(1):260. doi:10.1186/s12913-022-07655-6
10. Ericson O, Hjelmgren J, Sjovall F, Soderberg J, Persson I. The Potential Cost and Cost-Effectiveness Impact of Using a Machine Learning Algorithm for Early Detection of Sepsis in Intensive Care Units in Sweden. *J Health Econ Outcomes Res*. 2022;9(1):101-110. doi:10.36469/jheor.2022.33951

Supplementary Table 4. The univariate mixed-effects regression results

Supplementary Table 4.1 The univariate mixed-effects regression results with NMBs as the dependent variable

Variables	Coefficient	95% CI	P value
Dependent variable: NMB with one-time GDP as WTP			
**Study characteristics**			
**Disease domain**			0.518
Chronic disease management[a]	(Reference)		
Mental and neurological disorder[b]	421	[−378, 1,221]	0.302
Acute disease or infectious diseases or special purpose codes[c]	489	[−823, 1,800]	0.465
Neoplastic diseases	588	[−515, 1,690]	0.296
**Country income level**			**0.000**
Low or middle income	(Reference)		
High income	**897**	**[268, 1,526]**	**0.005**
**WHO geographical region**			
African region (AFR)	(Reference)		
Region of the Americas (AMR)	**1,192**	**[337, 2,046]**	**0.006**
South-East Asian region (SEAR)	37		
European region (EUR)	**873**	**[426, 1,320]**	**0.000**
Eastern Mediterranean region (EMR)	**74**	**[74, 74]**	**0.000**
Western Pacific region (WPR)	**538**	**[69, 1,007]**	**0.025**
**Target population—age**			**0.004**
All/not specified	(Reference)		
Pediatric	−631	[−1,296, 33]	0.063
Adult	−201	[−1,102, 701]	0.662
Senior	**−841**	**[−1,525, −156]**	**0.016**
**Target population—sex**			0.407
Mixed-sex/not specified	(Reference)		
All female/all male	−304	[−1,024, 415]	0.407
**Funder type**			0.548
Public or private-non-for-profit	(Reference)		
Private-for-profit	525	[−1,067, 2,118]	0.518
No/unspecified funding sources	−153	[−979, 674]	0.717
**Developmental stage at evaluation**			0.446
Market-access stage	(Reference)		
Early-stage	−296	[−1,058, 466]	0.446
**Intervention characteristics**			
**Type of AI-PM intervention**			**0.000**
Digital diagnostics	(Reference)		
Risk prediction	438	[−668, 1,543]	0.438
Precision treatment			
Disease self-management	**3,335**	**[2,797, 3,872]**	**0.000**
**Type of digital/electronic product**			0.607
Standalone algorithm	(Reference)		
Software or interactive app	−382	[−1,836, 1,073]	0.607
**AI-PM cost**	**3.63**	**[2.77, 4.48]**	**0.000**
**Integrated AI performance rate**			0.817
No	(Reference)		
Yes	−171	[−1,626, 1,283]	0.817

Variables	Coefficient	95% CI	P value
**Integrated adherence to AI technology**			0.342
No	(Reference)		
Yes	−247	[−755, 262]	0.342
**Integrated compliance with AI-informed decisions**			0.094
No	(Reference)		
Yes	−2321	[−5,038, 396]	0.094
**Model specifications**			
**Type of comparators**			**0.000**
Current practice/standard of care/none	(Reference)		
New technology/best alternative/major competitor	**−616**	**[−862, −369]**	**0.000**
**Applied any EE guidance**			0.6537
No	(Reference)		
Yes	−233	[−1,252, 786]	0.654
**Study perspective**			0.334
Societal	(Reference)		
Health system	−769	[−2,329, 790]	0.334
**Lifetime horizon**			0.845
No	(Reference)		
Yes	−106	[−1,168, 957]	0.845

[a] The "Chronic Disease Management" included Diseases of the Circulatory System, Diseases of the Digestive System, Diseases of the Genitourinary System, Diseases of the Musculoskeletal System or Connective Tissue.

[b] The "Mental and Neurological Disorder" included Diseases of the Nervous System, Mental, Behavioral or Neurodevelopmental Disorders, Sleep-Wake Disorders, Diseases of the Visual System.

[c] The "Acute Disease or Infectious Diseases or Special Purpose Codes" included Infectious or Parasitic Diseases, Codes for Special Purposes, Injury, Poisoning or Certain Other Consequences of External Causes.

Supplementary Table 4.2 The univariate mixed-effects regression results with Δcosts as the dependent variable

Variables	Coefficient	95% CI	P value
Dependent variable: Δcost			
**Study characteristics**			
**Disease domain**			0.491
Chronic disease management[a]	(Reference)		
Mental and neurological disorder[b]	−520	[−1,359, 319]	0.225
Acute disease or infectious diseases or special purpose codes[c]	−449	[−1,243, 346]	0.269
Neoplastic diseases	−174	[−696, 348]	0.514
**Country income level**			0.267
Low or middle income	(Reference)		
High income	−333	[−735, 69]	0.104
**WHO geographical region**			
African region (AFR)	(Reference)		
Region of the Americas (AMR)	−400	[−932, 133]	0.141
South-East Asian region (SEAR)	−9		
European region (EUR)	**−185**	**[−354, −16]**	**0.032**
Eastern Mediterranean region (EMR)	−57		
Western Pacific region (WPR)	−1	[−162, 159]	0.987
**Target population—age**			0.159
All/not specified	(Reference)		

Variables	Coefficient	95% CI	P value
Pediatric	113	[−388, 614]	0.658
Adult	69	[−540, 678]	0.825
Senior	**836**	**[22, 1,650]**	**0.044**
**Target population—sex**			0.646
Mixed-sex/not specified	(Reference)		
All male/all female	100	[−326, 525]	0.646
**Funder type**			**0.000**
Public or private-non-for-profit	(Reference)		
Private-for-profit	−793	[−1,634, 49]	0.065
No/unspecified funding sources	−16	[−246, 214]	0.893
**Developmental stage at evaluation**			0.512
Market-access stage	(Reference)		
Early-stage	185	[−369, 740]	0.512
**Intervention characteristics**			
**Type of AI-PM intervention**			**0.000**
Digital diagnostics	(Reference)		
Risk prediction	−493	[−1,282, 296]	0.221
Precision treatment			
Disease self-management	**−2760**	**[−2,890, −2,629]**	**0.000**
**Type of digital/electronic product**			0.365
Standalone algorithm	(Reference)		
Software or interactive app	526	[−613, 1,666]	0.365
**AI-PM cost**	**−2.68**	**[−3.04, −2.32]**	**0.000**
**Integrated AI performance rate**			0.434
No	(Reference)		
Yes	362	[−544, 1,267]	0.434
**Integrated adherence to AI technology**			0.149
No	(Reference)		
Yes	348	[−125, 820]	0.149
**Integrated compliance with AI-informed decisions**			0.702
No	(Reference)		
Yes	2429	[−10,021, 14,880]	0.702
**Model specifications**			
**Type of comparators**			0.373
Current practice/standard of care/none	(Reference)		
New technology/best alternative/major competitor	114	[−137, 366]	0.373
**Applied any EE guidance**			0.268
No	(Reference)		
Yes	263	[−202, 728]	0.268
**Study perspective**			0.780
Societal	(Reference)		
Health system	−82	[−658, 494]	0.780
**Lifetime horizon**			0.100
No	(Reference)		
Yes	493	[−94, 1,080]	0.100

[a] The "Chronic Disease Management" included Diseases of the Circulatory System, Diseases of the Digestive System, Diseases of the Genitourinary System, Diseases of the Musculoskeletal System or Connective Tissue.

[b] The "Mental and Neurological Disorder" included Diseases of the Nervous System, Mental, Behavioral or Neurodevelopmental Disorders, Sleep-Wake Disorders, Diseases of the Visual System.

[c] The "Acute Disease or Infectious Diseases or Special Purpose Codes" included Infectious or Parasitic Diseases, Codes for Special Purposes, Injury, Poisoning or Certain Other Consequences of External Causes.

Supplementary Table 4.3 The univariate mixed-effects regression results with ΔQALYs as the dependent variable

Variables	Coefficient	95% CI	P value
Dependent variable: ΔQALY			
**Study characteristics**			
**Disease domain**			0.558
Chronic disease management[a]	(Reference)		
Mental and neurological disorder[b]	0.012	[−0.008, 0.033]	0.240
Acute disease or infectious diseases or special purpose codes[c]	0.005	[−0.007, 0.017]	0.443
Neoplastic diseases	0.006	[−0.009, 0.021]	0.416
**Country income level**			0.470
Low or middle income	(Reference)		
High income	−0.009	[−0.034, 0.016]	0.470
**WHO geographical region**			
African region (AFR)	(Reference)		
Region of the Americas (AMR)	0.008	[−0.003, 0.018]	0.150
South-East Asian region (SEAR)	−0.0002		
European region (EUR)	**0.011**	**[0.003, 0.018]**	**0.006**
Eastern Mediterranean region (EMR)	0.010		
Western Pacific region (WPR)	0.029	[−0.004, 0.062]	0.090
**Target population—age**			**0.021**
All/not specified	(Reference)		
Pediatric	−0.026	[−0.058, 0.005]	0.104
Adult	−0.016	[−0.048, 0.017]	0.347
Senior	−0.020	[−0.055, 0.016]	0.279
**Target population—sex**			**0.092**
Mixed-sex/not specified	(Reference)		
All male/all female	−0.011	[−0.023, 0.002]	0.092
**Funder type**			0.242
Public or private-non-for-profit	(Reference)		
Private-for-profit	−0.008	[−0.026, 0.011]	0.403
No/unspecified funding sources	−0.013	[−0.031, 0.004]	0.140
**Developmental stage at evaluation**			0.226
Market-access stage	(Reference)		
Early-stage	−0.008	[−0.020, 0.005]	0.226
**Intervention characteristics**			
**Type of AI-PM intervention**			**0.041**
Digital diagnostics	(Reference)		
Risk prediction	−0.007	[−0.022, 0.007]	0.316
Precision treatment			
Disease self-management	0.001	[−0.011, 0.014]	0.831
**Type of digital/electronic product**			0.367
Standalone algorithm	(Reference)		
Software or interactive app	0.006	[−0.007, 0.019]	0.367
**AI-PM cost**	−4.54e-6	[−3.51e-5, 2.61e-5]	0.772
**Integrated AI performance rate**			0.070
No	(Reference)		
Yes	0.011	[−0.001, 0.024]	0.070
**Integrated adherence to AI technology**			**0.016**
No	(Reference)		
Yes	**0.054**	**[0.010, 0.099]**	**0.016**
**Integrated compliance with AI-informed decisions**			0.322

Variables	Coefficient	95% CI	P value
No	(Reference)		
Yes	−0.007	[−0.021, 0.007]	0.322
**Model specifications**			
**Type of comparators**			**0.025**
Current practice/standard of care/none	(Reference)		
New technology/best alternative/major competitor	**−0.015**	**[−0.028, −0.002]**	**0.025**
**Applied any EE guidance**			0.599
No	(Reference)		
Yes	−0.005	[−0.023, 0.013]	0.599
**Study perspective**			0.084
Societal	(Reference)		
Health system	−0.022	[−0.046, 0.003]	0.084
**Lifetime horizon**			0.918
No	(Reference)		
Yes	−0.001	[−0.019, 0.017]	0.918

[a] The "Chronic Disease Management" included Diseases of the Circulatory System, Diseases of the Digestive System, Diseases of the Genitourinary System, Diseases of the Musculoskeletal System or Connective Tissue.

[b] The "Mental and Neurological Disorder" included Diseases of the Nervous System, Mental, Behavioral or Neurodevelopmental Disorders, Sleep-Wake Disorders, Diseases of the Visual System.

[c] The "Acute Disease or Infectious Diseases or Special Purpose Codes" included Infectious or Parasitic Diseases, Codes for Special Purposes, Injury, Poisoning or Certain Other Consequences of External Causes.

Supplementary Figure 2. Cost-effectiveness profiles of AI-PM tools plotted against unit cost

A.

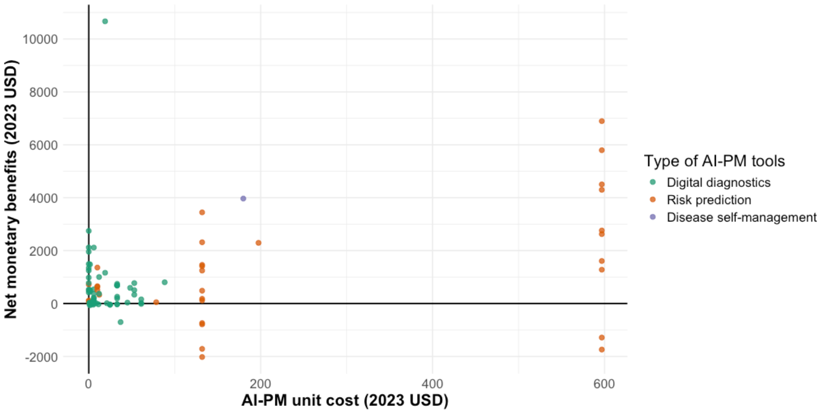

B.

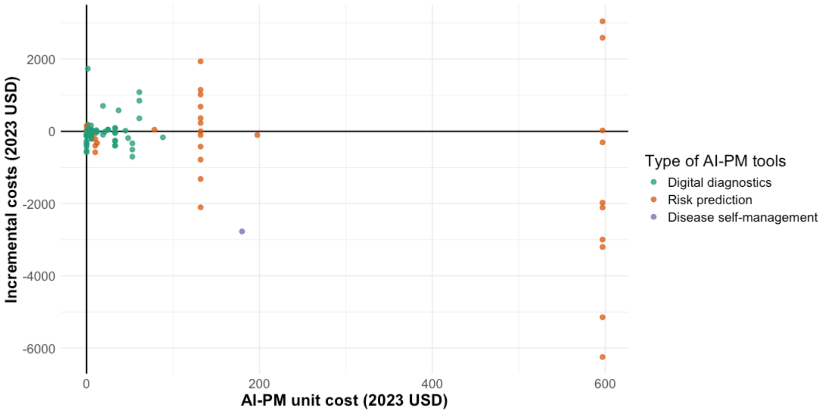

C.

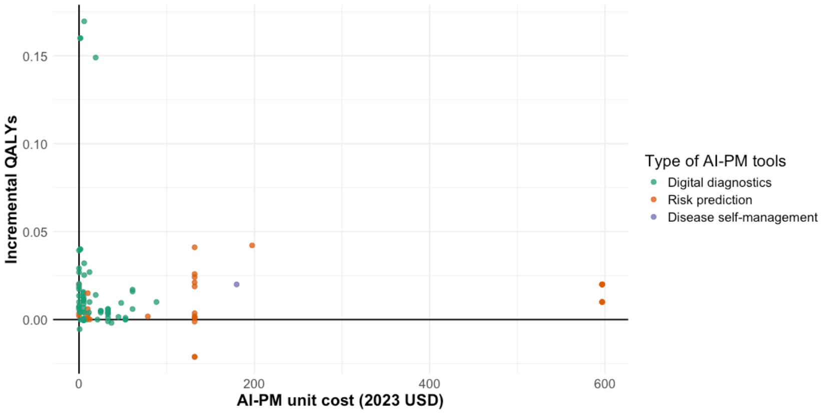

Supplementary Figure 2. Cost-effectiveness profiles of AI-PM tools plotted against unit cost. (A) Net monetary benefits plotted against AI-PM unit cost. (B) Incremental costs plotted against AI-PM unit cost. (C) Incremental quality-adjusted life-years plotted against AI-PM unit cost. *AI* artificial intelligence, *PM* precision medicine. Each dot represents an individual economic evaluation, colored by AI-PM type, including digital diagnostics, risk prediction, and disease self-management.

Supplementary Table 5. Preferred Reporting Items for Systematic Reviews and Meta-analyses (PRISMA) checklist

Section and Topic	Item #	Checklist item	Location where item is reported
**TITLE**			
Title	1	Identify the report as a systematic review.	1
**ABSTRACT**			
Abstract	2	See the PRISMA 2020 for Abstracts checklist.	2
**INTRODUCTION**			
Rationale	3	Describe the rationale for the review in the context of existing knowledge.	3-4
Objectives	4	Provide an explicit statement of the objective(s) or question(s) the review addresses.	4
**METHODS**			
Eligibility criteria	5	Specify the inclusion and exclusion criteria for the review and how studies were grouped for the syntheses.	14
Information sources	6	Specify all databases, registers, websites, organisations, reference lists and other sources searched or consulted to identify studies. Specify the date when each source was last searched or consulted.	14; Supplementary Table 6
Search strategy	7	Present the full search strategies for all databases, registers and websites, including any filters and limits used.	14; Supplementary Table 6
Selection process	8	Specify the methods used to decide whether a study met the inclusion criteria of the review, including how many reviewers screened each record and each report retrieved, whether they worked independently, and if applicable, details of automation tools used in the process.	14-15
Data collection process	9	Specify the methods used to collect data from reports, including how many reviewers collected data from each report, whether they worked independently, any processes for obtaining or confirming data from study investigators, and if applicable, details of automation tools used in the process.	15
Data items	10a	List and define all outcomes for which data were sought. Specify whether all results that were compatible with each outcome domain in each study	15

Section and Topic	Item #	Checklist item	Location where item is reported
		were sought (e.g. for all measures, time points, analyses), and if not, the methods used to decide which results to collect.	
	10b	List and define all other variables for which data were sought (e.g. participant and intervention characteristics, funding sources). Describe any assumptions made about any missing or unclear information.	15
Study risk of bias assessment	11	Specify the methods used to assess risk of bias in the included studies, including details of the tool(s) used, how many reviewers assessed each study and whether they worked independently, and if applicable, details of automation tools used in the process.	15
Effect measures	12	Specify for each outcome the effect measure(s) (e.g. risk ratio, mean difference) used in the synthesis or presentation of results.	15-16
Synthesis methods	13a	Describe the processes used to decide which studies were eligible for each synthesis (e.g. tabulating the study intervention characteristics and comparing against the planned groups for each synthesis (item #5)).	15
	13b	Describe any methods required to prepare the data for presentation or synthesis, such as handling of missing summary statistics, or data conversions.	16
	13c	Describe any methods used to tabulate or visually display results of individual studies and syntheses.	16
	13d	Describe any methods used to synthesize results and provide a rationale for the choice(s). If meta-analysis was performed, describe the model(s), method(s) to identify the presence and extent of statistical heterogeneity, and software package(s) used.	16-17
	13e	Describe any methods used to explore possible causes of heterogeneity among study results (e.g. subgroup analysis, meta-regression).	16-17
	13f	Describe any sensitivity analyses conducted to assess robustness of the synthesized results.	N/A
Reporting bias assessment	14	Describe any methods used to assess risk of bias due to missing results in a synthesis (arising from reporting biases).	15
Certainty assessment	15	Describe any methods used to assess certainty (or confidence) in the body of evidence for an outcome.	15
**RESULTS**			
Study selection	16a	Describe the results of the search and selection process, from the number of records identified in the search to the number of studies included in the	4; Figure 1

Section and Topic	Item #	Checklist item	Location where item is reported
		review, ideally using a flow diagram.	
	16b	Cite studies that might appear to meet the inclusion criteria, but which were excluded, and explain why they were excluded.	Figure 1
Study characteristics	17	Cite each included study and present its characteristics.	4-5; Table 1; Supplementary Table 1; Supplementary Table 2
Risk of bias in studies	18	Present assessments of risk of bias for each included study.	7-8; Figure 4
Results of individual studies	19	For all outcomes, present, for each study: (a) summary statistics for each group (where appropriate) and (b) an effect estimate and its precision (e.g. confidence/credible interval), ideally using structured tables or plots.	Figure 2
Results of syntheses	20a	For each synthesis, briefly summarise the characteristics and risk of bias among contributing studies.	4-5; 7-8; Table 1; Figure 4; Supplementary Table 2
	20b	Present results of all statistical syntheses conducted. If meta-analysis was done, present for each the summary estimate and its precision (e.g. confidence/credible interval) and measures of statistical heterogeneity. If comparing groups, describe the direction of the effect.	6-7; Figure 3; Table 2
	20c	Present results of all investigations of possible causes of heterogeneity among study results.	7; Table 2
	20d	Present results of all sensitivity analyses conducted to assess the robustness of the synthesized results.	N/A
Reporting biases	21	Present assessments of risk of bias due to missing results (arising from reporting biases) for each synthesis assessed.	7-8; Figure 4
Certainty of evidence	22	Present assessments of certainty (or confidence) in the body of evidence for each outcome assessed.	7-8, Figure 4
**DISCUSSION**			

Section and Topic	Item #	Checklist item	Location where item is reported
Discussion	23a	Provide a general interpretation of the results in the context of other evidence.	8-9
	23b	Discuss any limitations of the evidence included in the review.	12-13
	23c	Discuss any limitations of the review processes used.	12-13
	23d	Discuss implications of the results for practice, policy, and future research.	10-12
**OTHER INFORMATION**			
Registration and protocol	24a	Provide registration information for the review, including register name and registration number, or state that the review was not registered.	N/A
	24b	Indicate where the review protocol can be accessed, or state that a protocol was not prepared.	N/A
	24c	Describe and explain any amendments to information provided at registration or in the protocol.	N/A
Support	25	Describe sources of financial or non-financial support for the review, and the role of the funders or sponsors in the review.	18
Competing interests	26	Declare any competing interests of review authors.	18
Availability of data, code and other materials	27	Report which of the following are publicly available and where they can be found: template data collection forms; data extracted from included studies; data used for all analyses; analytic code; any other materials used in the review.	18

Supplementary Table 6. Search strategy and results

**Database: Embase and Medline**	
Search strategy	(((((('economic evaluation'/exp OR 'economic evaluation' OR 'cost effectiveness'/exp OR 'cost effectiveness' OR ((economic* NEAR/3 (evaluat* OR value)):ab,ti) OR (((cost OR costs) NEAR/3 (benefit* OR effectiv* OR efficien* OR efficac* OR minim* OR utilit* OR consequen*)):ab,ti)) AND ('economic model'/exp OR 'economic model' OR 'simulation'/exp OR 'simulation' OR (('model'/exp OR 'model') AND ('economics'/exp OR 'economics' OR 'economic aspect'/exp OR 'economic aspect')) OR 'decision tree'/exp OR 'decision tree' OR (((model OR modeling OR modelling OR simulation* OR microsimulation*) NEAR/6 (econom* OR pharmacoeconom* OR cost OR costs)):ab,ti) OR ((decision NEAR/3 (analy* OR tree OR trees)):ab,ti) OR 'discrete event*':ab,ti OR 'state transition':ab,ti OR markov:ab,ti OR (((individual* OR 'patient level*') NEAR/3 (sampl* OR simulation*)):ab,ti) OR ((dynamic NEAR/3 transmission*):ab,ti) OR probabilistic*:ab,ti OR 'partition* survival*':ab,ti)) AND ('artificial intelligence'/exp OR 'artificial intelligence' OR 'machine learning'/exp OR 'machine learning' OR 'deep learning'/exp OR 'deep learning' OR 'learning algorithm'/exp OR 'learning algorithm':ab,ti)) AND ('screening'/exp OR 'screening' OR 'diagnosis'/exp OR 'diagnosis' OR 'diagnostic'/exp OR 'diagnostic' OR 'risk'/exp OR 'risk' OR 'therapy'/exp OR 'therapy' OR (((personalized OR personalised OR individualised OR individualized OR precision OR stratif* OR targeted* OR algorithm*) NEAR/6 (screen* OR diagno* OR medicine* OR therap* OR treat* OR risk OR regimen* OR dosing* OR duration OR decision*)):ab,ti) OR ((model* NEAR/3 (guide* OR base*) NEAR/3 (screen* OR diagno* OR medicine* OR therap* OR treat* OR risk OR regimen* OR dosing* OR duration OR decision*)):ab,ti) OR ((risk NEAR/3 score*):ab,ti))) AND [2013-2023]/py) AND ([article]/lim OR [article in press]/lim)) AND [english]/lim AND ([embase]/lim OR [medline]/lim OR [preprint]/lim OR [pubmed-not-medline]/lim)
Results	1,539
Screenshot	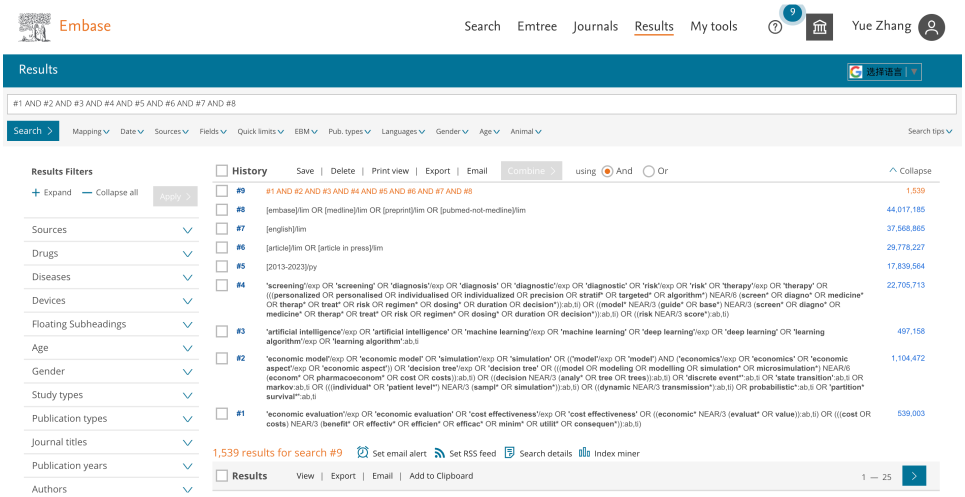
**Database: Web of Science**	
Search strategy	TS=("economic evaluation" OR "cost effectiveness" OR (economic* NEAR/2 (evaluat* OR value)) OR ((cost OR costs) NEAR/2 (benefit* OR effectiv* OR efficien* OR efficac* OR minim* OR utilit* OR consequen*)))<br>AND<br>TS=("economic model" OR simulation OR (model AND (economics OR "economic aspect")) OR "decision tree" OR ((model OR modeling OR modelling OR simulation* OR microsimulation*) NEAR/5 (econom* OR pharmacoeconom* OR cost OR costs)) OR (decision NEAR/2 (analy* OR tree OR trees)) OR discrete-event* OR "state transition" OR markov OR ((individual* OR patient-level*) NEAR/2 (sampl* OR simulation*)) OR (dynamic NEAR/2 transmission*) OR probabilistic* OR partition*-survival*)<br>AND<br>TS=((artificial* NEAR/3 intelligen*) OR (machine* NEAR/3 learning) OR (deep* NEAR/3 learning) OR (learning NEAR/3 algorithm))

	AND<br>TS=(screening OR diagnosis OR diagnostic OR risk OR therapy OR ((personalized OR personalised OR individualised OR individualized OR precision OR stratif* OR targeted* OR algorithm*) NEAR/5 (screen* OR diagno* OR medicine* OR therap* OR treat* OR risk OR regimen* OR dosing* OR duration OR decision*)) OR (model* NEAR/2 (guide* OR base*) NEAR/2 (screen* OR diagno* OR medicine* OR therap* OR treat* OR risk OR regimen* OR dosing* OR duration OR decision*)) OR (risk NEAR/2 score*))<br>AND<br>(DT=(article) OR DT=(article in press))<br>AND<br>LA=(english)<br>Publication Date: 2013-01-01 to 2023-11-03
Results	457
Screenshot	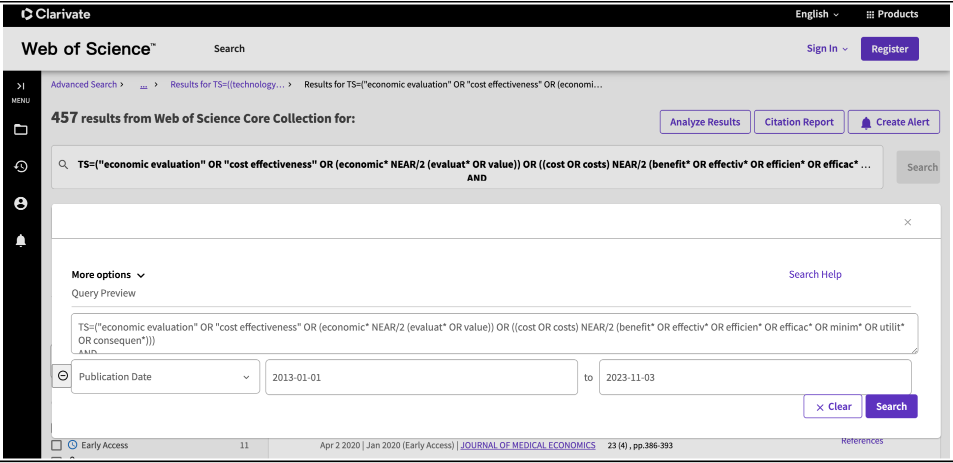
**Database: International HTA Database**	
Search strategy	((((personalized OR personalised OR individualised OR individualized OR precision OR stratif* OR targeted* OR algorithm*) NEAR6 (screen* OR diagno* OR medicine* OR therap* OR treat* OR risk OR regimen* OR dosing* OR duration OR decision*)) OR (model* NEAR3 (guide* OR base*) NEAR3 (screen* OR diagno* OR medicine* OR therap* OR treat* OR risk OR regimen* OR dosing* OR duration OR decision*)) OR (risk NEAR3 score*))[title]<br>OR<br>(((personalized OR personalised OR individualised OR individualized OR precision OR stratif* OR targeted* OR algorithm*) NEAR6 (screen* OR diagno* OR medicine* OR therap* OR treat* OR risk OR regimen* OR dosing* OR duration OR decision*)) OR (model* NEAR3 (guide* OR base*) NEAR3 (screen* OR diagno* OR medicine* OR therap* OR treat* OR risk OR regimen* OR dosing* OR duration OR decision*)) OR (risk NEAR3 score*))[abs]<br>(Mass Screening OR Diagnostic Screening Programs OR Diagnosis OR Therapeutics)[mh])<br>AND<br>(('artificial intelligence' OR 'machine learning' OR 'deep learning' OR 'learning algorithm')[title]<br>('artificial intelligence' OR 'machine learning' OR 'deep learning' OR 'learning algorithm')[abs]<br>(Machine Learning OR Artificial Intelligence OR Deep Learning OR Algorithms)[mh])<br>AND<br>* FROM 2013 TO 2023
Results	18

<table>
<tr><td>Screenshot</td><td>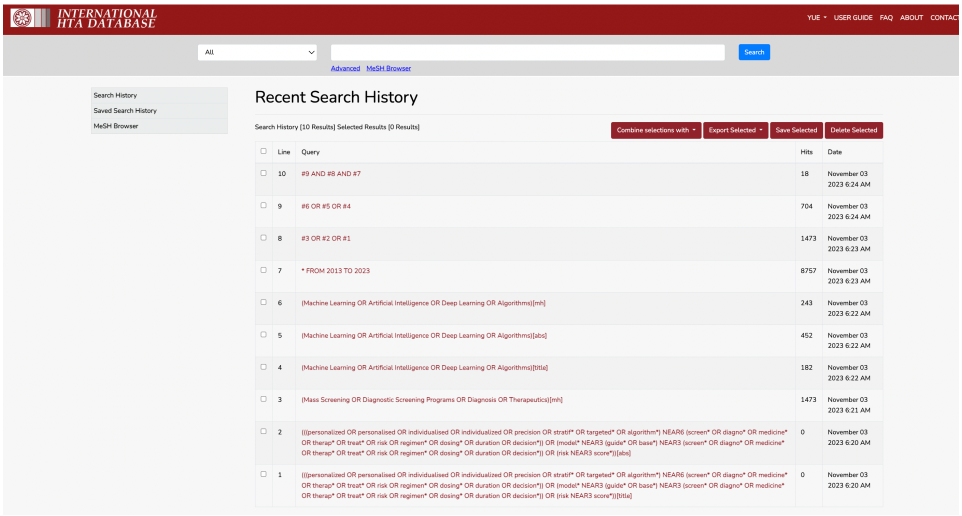
</td></tr>
<tr><td colspan="2">Database: Tufts Registry</td></tr>
<tr><td>Search strategy</td><td>(artificial intelligence)[abstract] OR (deep learning)[abstract] OR (machine learning)[abstract] OR (random forest)[abstract] OR (support vector machine)[abstract] OR (reinforcement learning)[abstract] OR (gradient boosting)[abstract] OR (neural network)[abstract] OR (natural language processing)[abstract] OR (computer vision)[abstract] OR (convolutional neural network)[abstract] OR (data mining)[abstract] OR (computer-aided detection)[abstract] OR (learning algorithm)[abstract] OR (risk algorithm)[abstract] OR (risk score)[abstract] OR (risk scores)[abstract]</td></tr>
<tr><td>Results</td><td>355</td></tr>
<tr><td>Screenshot</td><td>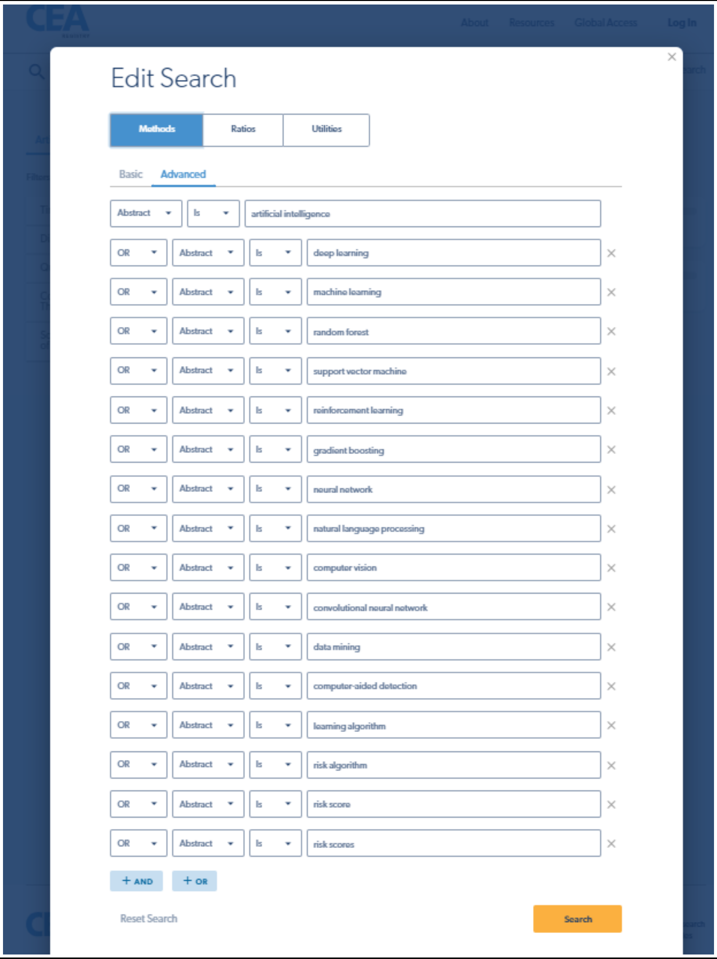
</td></tr>
</table>
